# Theory of Carbon Nanotube (CNT)-Based Electron Field Emitters

**DOI:** 10.3390/nano3030393

**Published:** 2013-07-17

**Authors:** Grigory S. Bocharov, Alexander V. Eletskii

**Affiliations:** 1 Moscow Power Engineering Institute, Technical University, Krasnokazarmennaya 14, Moscow 111250, Russia; E-Mail: bocharovgs@mail.ru; 2 National Research Center, Kurchatov Institute, Kurchatov sq. 1, Moscow 123182, Russia

**Keywords:** carbon nanotubes, electron field emitters

## Abstract

Theoretical problems arising in connection with development and operation of electron field emitters on the basis of carbon nanotubes are reviewed. The physical aspects of electron field emission that underlie the unique emission properties of carbon nanotubes (CNTs) are considered. Physical effects and phenomena affecting the emission characteristics of CNT cathodes are analyzed. Effects given particular attention include: the electric field amplification near a CNT tip with taking into account the shape of the tip, the deviation from the vertical orientation of nanotubes and electrical field-induced alignment of those; electric field screening by neighboring nanotubes; statistical spread of the parameters of the individual CNTs comprising the cathode; the thermal effects resulting in degradation of nanotubes during emission. Simultaneous consideration of the above-listed effects permitted the development of the optimization procedure for CNT array in terms of the maximum reachable emission current density. In accordance with this procedure, the optimum inter-tube distance in the array depends on the region of the external voltage applied. The phenomenon of self-misalignment of nanotubes in an array has been predicted and analyzed in terms of the recent experiments performed. A mechanism of degradation of CNT-based electron field emitters has been analyzed consisting of the bombardment of the emitters by ions formed as a result of electron impact ionization of the residual gas molecules.

## 1. Introduction

Carbon nanotubes (CNTs) have unique emission characteristics [1,2,3,4,5] which are caused by their high aspect ratio and good electrical conductivity. In combination with high thermal, mechanical and chemical stability, these properties of CNTs determine a possibility of development of a new type of vacuum electronic devices involved CNT-based electron field emission cathodes. In contrast with conventional electron field emitters, such cathodes operate at a relatively low applied voltage (at level 1 kV) which permits the development of miniature devices for wide application. This possibility is provided by electric field enhancement: due to this effect the electric field near a nanotube tip can be several hundred times higher than the average electric field strength in the interelectrode gap. A wide application of CNT-based cathodes and the prospects of their use in flat monitors [6,7,8], X-ray sources [9,10,11,12,13,14,15,16,17], lighting tubes [18,19,20,21,22,23], and the microwave radiation generators and amplifiers involved in the system of space communication [24,25] make it necessary to find the physical factors that limit the emission current density from such cathodes and to determine the maximum possible current density.

The *I*–*V* characteristic of an individual nanotube is described by the well-known Fowler–Nordheim relationship with an acceptable accuracy [26,27]. This relationship connects the emission current to the electric field strength near a CNT tip at a given cross section and the work function of the CNT. However, the *I*–*V* characteristic of a cathode containing a large number of CNTs can differ substantially from this relationship because of various effects. The main physical factors affecting the emission current density include the screening of an electric field by neighboring nanotubes, which decreases the electric field gain with increasing intertube distance [28,29,30]; thermal instability, that restricts the emission current from a nanotube [31]; and a statistical scatter of the individual parameters of a nanotube, which changes the character of the *I*–*V* characteristic of a cathode [30,31]. Some other effects should also be taken into account to analyze and optimize the operation of a CNT-based field emission cathode. The present article reviews theoretical efforts and approaches in theoretical analysis of physical effects responding for operation of CNT-based cathodes and determining their optimum characteristics in terms of the maximum reachable electron emission current density, lifetime, *etc*. Note that the current status of research on development, fabrication procedure and application achievements has been reviewed in detail in [5,32]. These review articles are mainly addressed to experimental aspects of the field and applied issues. In distinction of those, the present review is devoted to theoretical methods and approaches applied in theoretical research related to CNT-based field emission cathodes. For completeness the survey has been supplemented with some results that have been already published earlier in various publications and reviewed in [5].

One should realize that carbon nanotubes produced by conventional methods are very spread in their physical properties. Electrical characteristics of single walled nanotubes (SWNT) depend considerably on their chirality and diameter [33,34]. The nanotubes having “armchair” structure possess metallic electrical properties; the rest ones have semiconductor characteristics. Therefore, only one third of nanotubes are good conductors of electricity. The forbidden band gap of semiconductor SWNTs is rather narrow (less 1 eV) and inversely proportional to their diameter, so that their conductivity at a temperature of 300–1000 K is quite high to provide the electron emission under the action of the electrical field. Taking into account these peculiarities, one can believe that all the SWNTs comprising the emission array contribute to the emission current while the contribution of various nanotubes can be different. The structure of multi-walled nanotubes (MWNT) is an assembly of SWNTs coaxially stacked one into another. The diameter of external nanotubes comprising MWNTs is quite high so that the forbidden band gap is negligible which allows one to assign them metallic conductivity. Therefore in further considerations the carbon nanotubes comprising the electron field emission cathodes will be believed as metallic. The influence of the natural spread in the electrical properties of CNTs onto the emission characteristics of the relevant cathode will be analyzed within the frame of the theoretical approach given in Section 3.2.

## 2. Emission Properties of an Individual Nanotube

### 2.1. Electron Field Emission and the Fowler-Nordheim Equation

The phenomenon of electron field emission is based on the effect of quantum tunneling of electrons found inside a ground conductor through a barrier that is formed by the ionic lattice of the conductor and the external electric field [26,27]. A simple quantum-mechanical approach [26,27] results in the following dependence of the emission current density *J* on the electric field strength *E*, called the Fowler-Nordheim (FN) Equation:

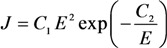
(1)

Here the parameters *С*_1_ and *С*_2_ are expressed through the magnitude of the electron work function *φ* for the conductor under consideration and the basic constants (the charge and the mass of electron *e* and *m* and the Plank constant *h*):

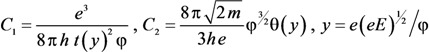
(2)

The functions *t*(*y*) and *θ*(*y*) present smoothly changing dependencies that can be well approximated by the relations *t*(*y*) ≈ 1 and *θ*(*y*) ≈ 1 – *y*^2^. The total emission current *i* is determined by the integration of Equation (1) over the emitting surface: 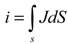.

The Fowler-Nordheim Equation (1) has an approximate character. This relation corresponds to the 1D situation, when the emitting surface of a conductor presents an infinite plane oriented in perpendicular to the direction of the external electrical field. Beside this relation has been derived assuming that all the electrons of conductivity in the emitter have the similar energy corresponding to the Fermi level of the material. This assumption is equivalent to the supposition that the temperature of the conductor is negligible comparing to the Fermi level (or the work function *φ*). In the case of violation of this supposition the energy of electrons capable to emit can be different which results in a temperature dependence of the emission current that can be taken into account using the relevant correction to the FN equation [27,35]. Beside of that it is assumed that the electron work function of the emitter does not depend on the orientation of the electrical field relating to the crystal axes of the conductor. Apparently the validity of this assumption in respect to carbon nanotubes was not yet studied. However, the spread in the values of the electron work function for CNT of various structures measured in different conditions [1] indicates the existence of such dependence.

One more factor complicating the character of the dependence (1) is the Shottky effect that manifests itself in a lowering of the potential barrier formed by the external electrical field due to the interaction of the electron escaping from the surface of the emitter with its mirror reflection. This effect is accounted by the correcting function *θ*(*y*) specified in Equation (2) which is notable at high fields. In the case of CNTs, whose electron work function is about *φ* ≈ 5 эВ, this correction is significant at the electrical field strength of order or higher than 10^8^ V/cm. Usually the value of this correction is within the uncertainty related to such factors as structural defects in CNT changing its electron characteristics, dependence of the work function of the orientation of the CNT, *etc*. These factors will be considered in detail below.

One should be noted that the Fowler-Nordheim Equation (1) relates to the flat geometry conductors. In this case the electron emission problem is 1D one. The real emitters and specifically CNTs are characterized by a considerably more complicated structure. A nanotube promotes a significant distortion of the electrical field in a vicinity of its tip. Therewith the magnitudes of the electrical field strength acting to various sites of the tip are differed from each other. In this connection there arises a problem of evaluation of the emission current of a CNT *vs.* the applied voltage with taking account the real geometry of the emitter and real distribution of the electrical field in its vicinity. This problem was stated by many authors (see, e.g., [36,37,38,39,40,41,42,43,44]). The calculations of such a kind promote a deeper understanding of the mechanism of the electron field emission of CNTs. However one should be noted that the degree of uncertainty of the geometry of both electrical field and CNTs comprising the emitter array, dependence of emission characteristics on the sort and quantity of adsorbed molecules and a considerable non-controllable spread in electronic properties of CNTs promote an uncertainty in their emission characteristics. This uncertainty is out of the corrections to the FN Equation (1) related to the above-cited approaches. This can be seen in particular from the comparison of measured current-voltage characteristics of an individual CNT with those calculated by Equation (1) which demonstrates a good agreement within a wide range of the emission current. Such an agreement allows one to utilize Equation (1) as a quite convenient starting point for analysis of experimental data concerning CNT-based electron field emitters. The physical mechanisms causing notable violation of this relation will be analyzed in detail below.

A convenient way to treatment and analysis of experimental data on the basis of FN Equation (1) consists in a logarithm representation of this relation which results in a linearity between the ratio *J*/*E*^2^ (or *i*/*E*^2^) and an inverse magnitude of the electrical field strength 1/*Е*:

ln(*i/E*^2^) = *C*_1_ – (*C*_2_/*E*) (3)

The rectilinear shape of this dependence indicates the mechanism of the electron emission relating to the electron field emission effect. The parameters of this dependence such as its slope and the points of crossing with the axes permit, in principle, determination of the area of the emitting surface and the electron work function.

**Figure 1:**
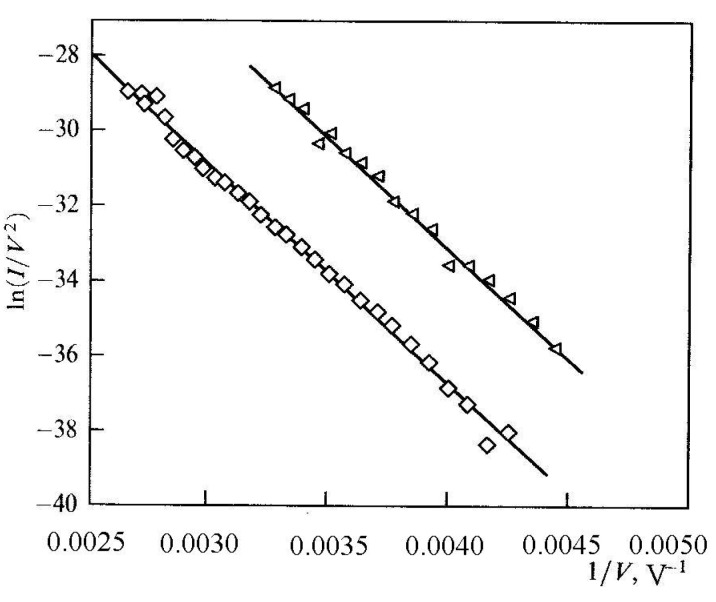
Typical current-voltage characteristics of an individual emitter on the basis of a multiwalled nanotube 8 nm in diameter and 1.1 mm in length, measured before (triangles) and after (rhombi) thermal treatment [45].

The results of numerous experiments imply that the emission properties of an individual CNT are described quite well by Equation (1). This is illustrated by the current-voltage characteristics of an individual CNT measured in [45] by means of the scanning electron microscope before and after thermal treatment (Figure 1). As is seen while the FN Equation (1) is rather approximate one, the current-voltage characteristics have a linear shape within quite wide region of the applied voltage. Therefore, this equation describes experimental data quite well. Possible deviations of measured current-voltage characteristics on the FN equation are always reasoned physically, which will be analyzed in detail below.

### 2.2. Electric Field Enhancement

#### 2.2.1. The Field Enhancement Effect and the Aspect Ratio of CNTs

The average value of the electrical field strength within the inter-electrode space *E_o_* is equal to the voltage *U* applied between anode and cathode divided by the inter-electrode distance *L*. If the region contains some spatial non-homogeneous features, the homogeneous distribution of the electrical potential in a vicinity of these features is distorted, so the electrical field strength in this region can exceed sufficiently the above-mentioned average value. The distortion can be characterized by the electrical field enhancement factor *β* defined as the ratio of the real electrical field strength to the average one:

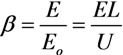
(4)

**Figure 2:**
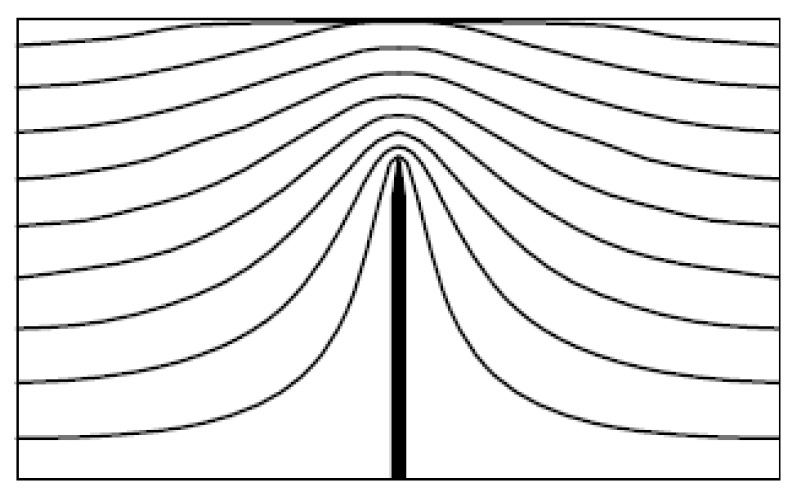
The typical spatial distribution of the electrical field potential in the vicinity of a sharp tip [46].

The effect of the electrical field enhancement is illustrated by Figure 2 reporting the typical shape of the distribution of the electrical field potential in the vicinity of a sharp tip. This distribution has been calculated through the solution of the Laplace equation for a region experience to the action of the applied potential [46].

A basic geometrical parameter determining the field enhancement factor of a single nanotube is its aspect ratio *α* = *h*/*d*, where *h* and *d* are the height and diameter of the nanotube, respectively. A rough estimation following from a qualitative analysis of the electrostatic problem yields a linear relationship between the field enhancement factor and the aspect ratio:

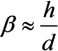
(5)

Since the aspect ratio for CNTs may reach 1000 or more, the field emission from nanotubes may take place at a much lower applied voltage than in the case of conventional field emitters.

**Figure 3:**
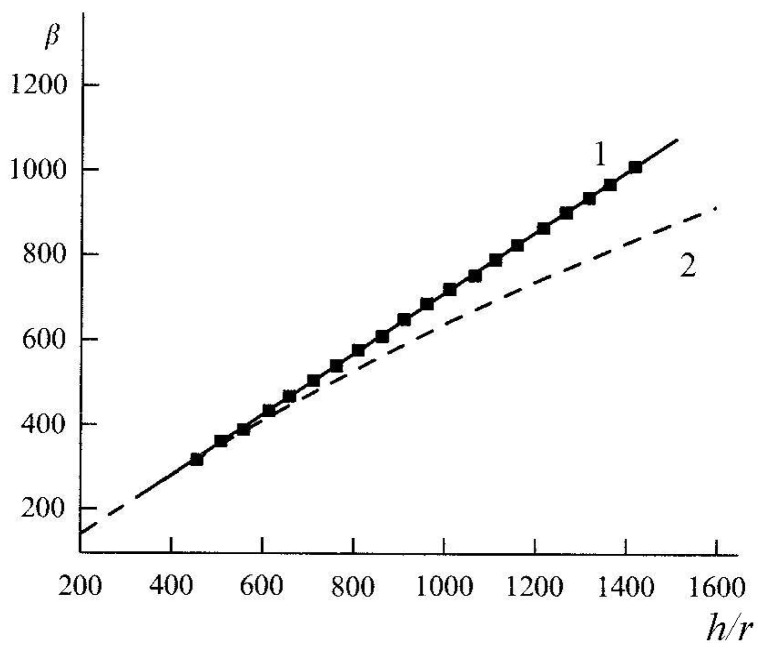
Field enhancement factor of a nanotube *vs*. its aspect ratio. The dependences approximated by (**1**) *β* = 0.31 + 0.71*h*/*r* [47], and (**2**) *β* = 5.93 + 0.73*h*/*r* − 0.001(*h*/*r*)^2^ [48].

The quantitative dependence of the field enhancement factor on the nanotube geometry and interelectrode spacing may be found by solving the electrostatic problem. The solution of this problem consists in solving the Laplace equation for the case when the potential on the cathode surface is zero and that on the anode surface is given. By solving this equation numerically, one can find the electric field strength throughout the interelectrode gap and then find the field enhancement factor according to expression in Equation (4). As an example of such a calculation, Figure 3 plots the dependences of field enhancement factor *β* on the aspect ratio that were obtained in [47,48]. The nanotube was simulated by a vertical flat-cap cylinder [47] and by a column consisting of stacked conductive spheres [48]. The somewhat different shape of the curves at high aspect ratios (see Figure 3) can be caused by different approaches to CNT simulation.

**Figure 4:**
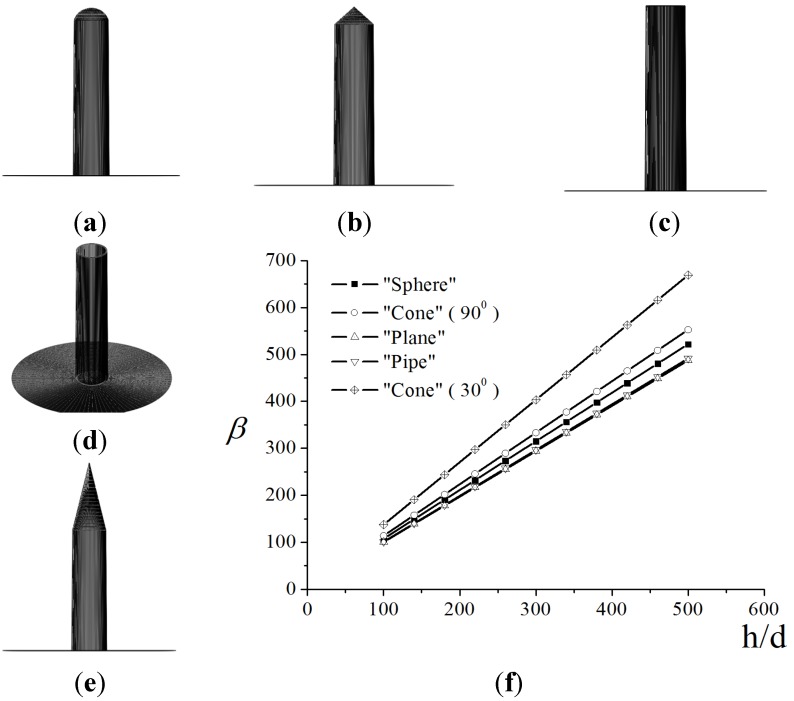
(**a**)–(**e**) Various shapes of the carbon nanotube (CNT) tip for which the field enhancement factor versus the aspect ratio was calculated. Curves **1***–***5** in panel (**f**) correspond to panels (**a**)–(**d**). The inter-electrode spacing and the applied voltage are 200 μm and 1000 V, respectively [49].

The “sensitivity” of the field enhancement factor *β* to the shape of the nanotube tip can be estimated from the comparison of the *β* *versus* the aspect ratio dependences taken at different tip shapes [49]. The calculations were carried out for a variable-height nanotube of 10 nm in diameter, an interelectrode spacing of 200 μm, and an applied voltage of 1000 V. Figure 4a–e show five shapes of tips for which the calculations were performed: (a) hemisphere, (b) cone with a cone angle of 90°, (c) flat cap, (d) open hollow cylinder with a 1 nm thick wall, and (e) cone with an angle of 30°. The calculation results are shown in Figure 2f.

It is seen that a change in the tip shape causes a change in the field enhancement factor within 5%–7%. The change is most significant in the case of the cone with an angle of 30°. The tip with such a shape increases the field enhancement effect.

One should note that the more detailed numerical calculations of the field enhancement factor in vicinity of an elongated vertically aligned cylindrical rod with different shape of the top have been performed by the authors of [50] who have determined the distribution of the factor over the top surface. The results obtained are the most valuable for macroscopic emitters of a simple structure, while in the case of CNT-based cathodes having much more complicated atomic-scale structure such an approach seemly is beyond of the possible accuracy of consideration. For this reason we restricted our consideration to rather roughly averaged structures of the tip for the sake of illustration of influence of the tip shape onto the field enhancement factor.

#### 2.2.2. Field Enhancement at Short Interelectrode Spacings

The above-given calculation results were obtained under the assumption that interelectrode spacing *L* far exceeds the height *h* of the nanotube. With this condition violated, the field enhancement factor becomes dependent on the interelectrode spacing. Indeed, if distance *D* between the flat tip of the nanotube and the anode surface is much smaller than the diameter *d* of the nanotube, the space between the anode and nanotube can be considered as a plane capacitor and the electric field strength *E* at the tip is given by the simple formula *E* = *U*/*D*. Since the mean electric field in the device is *E_o_* = *U*/(*h* + *D*), the field enhancement factor can be expressed as:

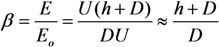
(6)

In the limit *D* >> *d*, the enhancement factor is seen to be independent of the nanotube diameter: it depends only on the ratio between the nanotube height and interelectrode distance. In the general case of an arbitrary relationship between *D* and *d*, the field enhancement factor certainly depends on the interelectrode spacing in a more complicated way and involves the nanotube diameter as a parameter.

With parameters *h*, *d*, and *D* taken arbitrarily, the following interpolation relationship between the geometry of the system and the electric field enhancement factor can be suggested:

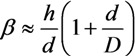
(7)

In the limits *D* >*>* *d* and *D* *<<* *d*, this expression turns into Equations (5) and (6), respectively. For the avoidance of confusion, it should be noted that different authors apply different approaches to determine the field enhancement factor. The standard approach [4] described by Equation (4) defines the field enhancement factor as a ratio between the electric field strength at the tip of the nanotube and the electric field in the nanotube-free gap. Alternatively [51,52,53], the enhancement factor is defined as a ratio between the electric field strength at the tip of the nanotube and the electric field at the anode surface. Both approaches yield the same values of *β* when the interelectrode spacing is large (*D* *>>* *d*). However, if this inequality is invalid, the field enhancement factors calculated in terms of these approaches differ appreciably. For *d* *>>* *D*, the alternative approach leads to the paradoxical conclusion that the field enhancement effect is absent (*β* = 1). At the same time, the field strengths at the tip calculated in terms of both approaches coincide; therefore, the alternative definition of the field enhancement factor should be considered as being inconvenient to analyze the situation rather than as being incorrect.

**Figure 5:**
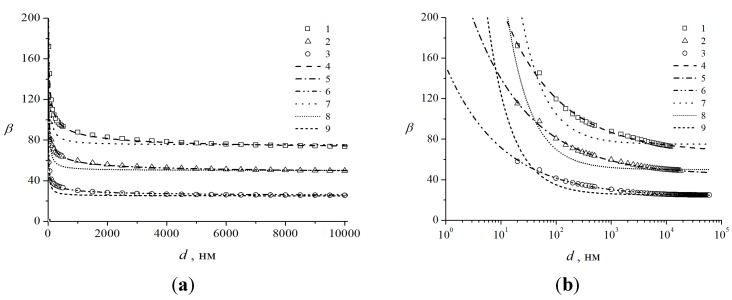
Field enhancement factor *β* *vs*. the distance between the nanotube top and anode surface for nanotubes of 400 nm in diameter with different heights. (□, Δ, ○ for *h* = 1, 2 and 3 μm, respectively). Calculations for nanotubes 3, 2, and 1 μm high, respectively, by approximant (8) (points **1**–**3**) and interpolation Equation (7) (points **4**–**6**). (**a**) Cartesian and (**b**) semilog coordinates.

The dependences of the field enhancement factor on the interelectrode spacing that were calculated by us for individual nanotubes with the above diameter and heights *h* = 1, 2, and 3 μm (corresponding aspect ratios *α* = *h*/*d* = 40, 50, and 75, respectively) are shown in Figure 5. The applied voltage in the calculations was set equal to 100 V. It is seen that the field enhancement factor starts depending on the interelectrode spacing even at *D*/*h* < 5 and; at *D* ~ *h*, the enhancement factor is roughly 1.5 times larger than its asymptotic value corresponding to large interelectrode spacings.

Xu *et al*. [51,54], experimentally and theoretically studied the field enhancement factor of the nanotube as a function of the geometrical factors of the system at short interelectrode spacings. They measured the field enhancement factor for a vertical nanotube 40 nm in diameter versus the interelectrode spacing. The numerical results of Xu *et al.* are well fitted by the approximant:

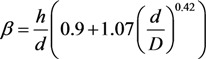
(8)
which, however, fails in the limit *D* *<<* *d* [in contrast to Equation (7)].

#### 2.2.3. Field Enhancement in the Case of Tilted Nanotubes

Above, we considered the field enhancement factor of vertically oriented nanotubes. Actually, however, nanotubes constituting an emitter array may be inclined to the cathode surface at different angles. This influences both the field enhancement factor of separate nanotubes and the *I*–*V* characteristic of the cathode as a whole. Below, we present the results of solving the electrostatic problem and draw a conclusion about the sensitivity of the CNT-based cathode’s emission properties to the deviation of nanotubes from the vertical.

The electric field spatial distribution at the tip of the nanotube tilted to the cathode surface at different angles was calculated in [55] by the boundary element method that is based on Green’s integral, which allows the determination of the potential at a given point through special integrals taken over the electrode surface [55,56]. In these integrals, the expression under the integral sign contains the product of an unknown function that is determined by solving integral equations and a fundamental solution to the Laplace equation that, in the 3D, equals unity divided by the distance from a given point to the center of a coordinate system chosen arbitrarily. In this way, the desired potential can be represented as a superposition of potentials produced by virtual charges placed on the surface of a conductor. The arrangement of these charges on the surface is selected so as to provide the potential spatial distribution in the region where this distribution is known. This approach ensures the adequate behavior of the potential at distances from the emitter surface that exceed the distance between nearby virtual charges. In the region near the emitter surface, the potential distribution is determined by extrapolation from a remote region.

**Figure 6:**
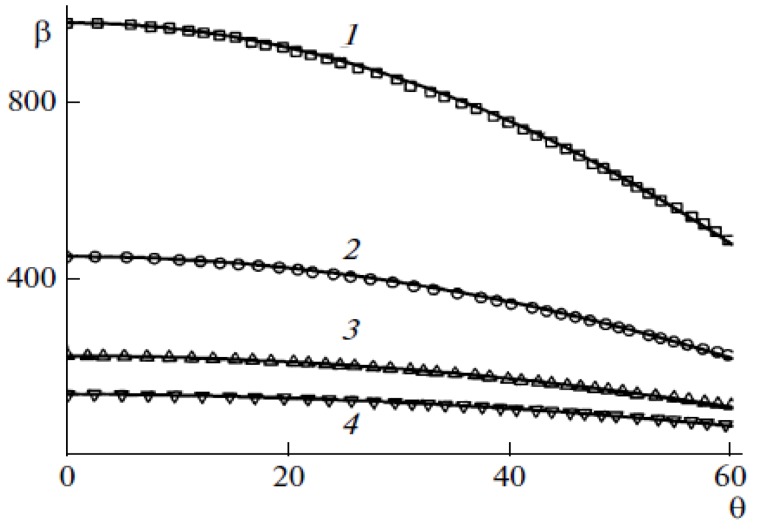
Field enhancement factor *vs*. the angle of tilting the nanotube to the cathode surface calculated for nanotubes of 1 μm in height and of 1.4 (**1**), 3.0 (**2**), 6.0 (**3**), and 10.0 (**4**) nm in diameter. Solid lines depict the parabolic approximation of the results calculated by Equation (9) [30].

Figure 6 plots the calculated dependences of the field enhancement factor on the inclination of 1 μm high nanotubes with different diameters. The curves are well approximated by the parabolic formula:

*β* = *β_0_*(1 – *kθ*^2^) (9)

where *θ* is the tilting angle of the nanotube. The values of field enhancement factor *β_0_* for a vertical nanotube and the fitting parameter *k* are given in the Table 1. As is seen the fitting parameter *k* is practically independent of the nanotube diameter.

**Table 1:** Values of the parameters figuring in relationship of Equation (9) for 1 μm high CNTs with different diameters.

*d* (nm)	1.4	3	6	10
*β*_0_	795	393	209	132
*k*	0.466	0.466	0.466	0.463

### 2.3. Thermal Effects

#### 2.3.1. Heat Conduction Equation

An increase in the temperature of a nanotube as a result of its Ohmic heating during emission can change its emission properties. These changes can be reflected in both the transport characteristics of the nanotube (electrical conductivity and thermal conductivity) and its emission capacity. Indeed, a conductor heated to high temperature can emit electrons at very low applied voltages (thermionic emission). This phenomenon is based on the existence in the conductor of high-energy electrons that are able to overcome the potential barrier formed by the ionic lattice. Therefore, one can expect a transition from electron field emission to thermionic emission with a rise in the emission current. Such a phenomenon is observed in CNT-based emitters, and the range of parameters where such a transition occurs is quite wide.

The Ohmic heating of a nanotube during the emission promotes a thermal inhomogeneity along its length, which can result in the thermal decomposition of the nanotube. This effect limits the maximum reachable emission current and determines the limiting operational characteristics of the relevant cathode. The elongated shape of a nanotube hinders the removal of heat from the nanotube to the substrate. This promotes the development of thermal instability which results in violation of the thermal balance in nanotubes emitting electrons. The concept of the thermal instability of a CNT-based emitter was formulated in [57], where it was shown that the heat conduction equation for a nanotube emitting electrons has a solution only within a limited range of variability of the emission current. Exceeding some critical magnitude of the emission current causes unlimited heating of the emitter, which is accompanied by its thermal decomposition. The physical reason for developing the thermal instability limiting the emission current of the nanotube is the sharp (exponential) character of the dependence of the emission current on the applied voltage. This exponential dependence is reflected in the dependence of the rate of heat release on the applied voltage. Since the dependence of the rate of heat removal on the applied voltage is not so strong, exceeding some critical value of the emission current leads to the violation of the thermal balance in the nanotube, which is accompanied by its thermal decomposition.

The thermal balance in a nanotube, taking into account Ohmic heating, radiation cooling, and heat removal by thermal conductivity, is described by the steady-state heat conduction equation having the following form [57,58]:

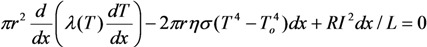
(10)

Here, the origin of coordinates (*x* = 0) is found at the point of contact between the nanotube and the substrate; *T* = *T*(*x*) is the temperature profile along the nanotube’s axis; *λ*(*T*) is the longitudinal thermal conductivity coefficient of the nanotube; *R*(*T*) is the longitudinal electrical resistance of the nanotube; *σ* is the Stephan-Boltzmann constant; *η* < 1 is the grayness coefficient, *r* is the external radius of the nanotube; *I* is the emission current. The first boundary condition corresponds to the requirement of the equality of the temperature in the point (*x* = 0) to the substrate temperature *T*_0_:

*T* (0) = *T*_0_ (11)

The second boundary condition is formulated assuming that the nanotube is found in a vacuum, so that the heat flux from the nanotube’s tip is zero. This corresponds to the following condition

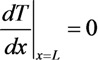
(12)

The emission current *I* involved in Equation (10) as a parameter is interconnected with the applied voltage through the Fowler-Nordheim Equation (1).

#### 2.3.2. Transport Coefficients

For the solution of Equation (9) one needs to have reliable data on the temperature dependences of thermal conductivity *λ*(*T*). and electrical resistance *R*(*T*) along the nanotube’s axis. At the present time, such available data are incomplete and contradictory, so that they are characterized by a spread within several orders of magnitude (see review [59] devoted to the transport properties of CNTs). Thus, the values of the thermal conductivity coefficient measured in [60,61] amount to 3000 and 25 W/mּK, respectively. The electrical resistance of a multiwalled CNT about 100 nm in length varies between 1.5 and 37 kΩ [62]. Investigations indicate that both transport coefficients of CNTs and their temperature dependences are determined by synthesis conditions and can vary within quite a wide range. The reason for such a spread is the occurrence of noncontrollable structural defects in CNTs, as well as adsorbed functional groups having a considerable influence on the electronic structure of the nanotube, phonon spectrum, and other characteristics determining CNT transport properties. Because of the above-mentioned uncertainty, it is hardly possible to obtain the accurate magnitude of the limiting temperature for the emitting nanotube on the basis of Equation (10).

A convenient approach for representing the temperature dependences of the electrical and thermal conductivities of CNTs is based on the so-called quasiballistic model of transport [59], according to which the charge is carried mainly by electrons, while the heat is transferred mainly by phonons. It being assumed that the ballistic mechanism of charge (heat) transport occurs for a section of a nanotube, whose length *l* corresponds to the mean free path *l*_e;p_ of the electron (phonon) relating to the elastic scattering on defects or admixtures. According to the ballistic mechanism, electrons (phonons) pass this section of the CNT without scattering, and the relevant values of quanta of the ballistic electrical conductivity *G*_0_ and phonon thermal conductivity *G*_th_ are expressed through the following equations:

*G*_0_ = 2*e*^2^ / *ћ* = 7.72 × 10−5 Ом−1 (13)

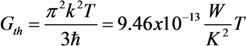
(14)

Here, *G*_th_ is the quantum of the phonon thermal conductivity in the limiting case when the characteristic energy of the quantum of phonon vibrations is much less than the temperature *T*. For obtaining the ballistic thermal conductivity of a CNT, one should multiply *G*_th_ by the total number of phonon channels *N*_p_ in the nanotube. The latter is a triple number of atoms in a unit cell, 2*N*, where *N* is expressed through the chirality indices (*n*; *m*) of the nanotube in the following way [8,9]:

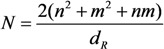


Here *d*_R_ is the greatest common divisor of (2*n* + *m*) and (2*m* + *n*). For a single-walled CNT with the *armchair* structure and the chirality indices (*n*, *n*) *d*_R_ = *n* and *N* = 6*n*. For example, a single-walled (10, 10) CNT (diameter 1.4 nm) has *N*_p_ = 120 phonon channels, while a (200, 200) CNT (diameter 27.5 nm) has *N*_p_ = 2400 phonon channels. Therefore, the ballistic thermal conductance of (10, 10) and (200, 200) CNTs amounts to 120*G*_th_ and 2400*G*_th_, respectively.

Usually the nanotube’s length *L* exceeds the characteristic value of the electron (phonon) mean free path *l*_e,p_ relating to the scattering on structural defects by several times or even by the order of magnitude. In this case corresponding to the quasi-ballistic regime of charge and heat transport the above-given expressions for the ballistic transport should be supplemented with a correcting factor:

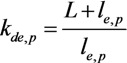
(15)

Taking account this correction the nanotube’s resistance is represented in the form:

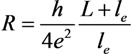
(16)
while the thermal conductivity is given as follows [59]:

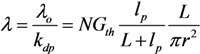
(17)

According to Equations (16) and (17), the transport coefficients of a CNT depend on its length, which can be considered as one of the manifestations of the dimension effect inherent to nanoobjects.

The above-presented consideration shows that the temperature dependences of transport coefficients within the framework of the quasiballistic mechanism of charge and heat transfers are determined by the relevant dependences of the electron (phonon) mean free path relating to the elastic scattering. The type of structural defects in CNTs determining the character of scattering of electrons and phonons is usually unknown, so that experiment serves as the main source of reliable data on the temperature dependences of transport coefficients [59]. In calculations, the equality *k*_de_ = *k*_dp_ is usually accepted, which corresponds to the equality between the electron and phonon mean free paths relating to the scattering on defects and other inhomogeneities. Because of the absence of reliable data on the temperature dependences of transport coefficients, these dependences are usually modeled by a power function:

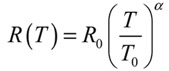
(18)
where *R*_0_ is the resistance at *T* = *T*_0_, and *α* is the fitting parameter. A similar approach is used for representing the temperature dependence of the thermal conductivity coefficient; however, taking into account the assumption *k*_de_ = *k*_dp_, the parameter *a* has the opposite sign in this case.

#### 2.3.3. Thermal Instability of a CNT-Based Emitter

**Figure 7:**
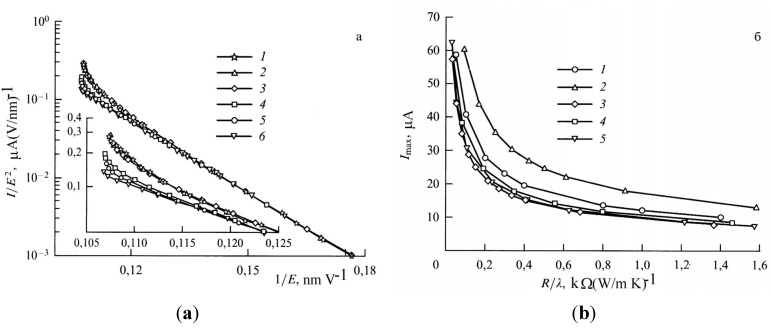
Results of the solution of the heat conduction Equation (10) in combination with the Fowler-Nordheim Equation (1) [57]: (**a**) *I*–*V* characteristics of a carbon nanotube 5 nm in radius and 1.6 mm in length calculated for various model temperature dependences of transport coefficients: **1**: experiment [63]; **2**: *α* = 4; **3**: *α* = −1; **4**: *α* = 0; **5**: *α* = 0; *λ* = const; **6**: *α* = 1. The inset presents an enlarged part of the current-voltage characteristics. (**b**) Dependences of the emission current *I*_max_ on the ratio *R/λ* calculated for various model temperature dependences of transport coefficients: **1**: *α* = 0, *λ* = const; **2**: *α* = 4; **3**: *α* = −1; **4**: *α* = 0; and **5**: *α* = 1.

Figure 7 presents the results of the solution of the heat conduction Equation (10) in combination with the Fowler-Nordheim Equation (1), obtained for a nanotube 5 nm in radius and 1.6 mm in length using various assumptions about the temperature dependences of transport coefficients [57]. The calculations indicate that, independently of those assumptions, there exists a maximum reachable emission current whose exceeding results in the development of thermal instability and thermal decomposition of the nanotube. Note that the calculations imply that, even assuming *η* = 1, the contribution of radiation losses to the thermal balance of a nanotube does not usually exceed 10%. One can expect that this contribution should increase as the nanotube’s length increases. Therefore, taking into account the radiation cooling of a nanotube does not influence qualitatively the above-formulated conclusion on the mechanism of limitation of the emission current, related to thermal instability. The calculated *I*–*V* characteristics of a nanotube 5 nm in radius and 1.6 mm in length with a field amplification factor of *β* = 218.1 and emitting surface area of *S*_em_ = 12.57 nm^2^ are compared in Figure 7a with those measured in [63] for a nanotube of the same geometry.

At low currents, the *I*–*V* characteristics in the FN coordinates have a rectilinear shape which corresponds to the classical Fowler-Nordheim dependence. Deviations of the *I*–*V* characteristic from this dependence, observed at high emission currents, are caused by the influence of heating the emitter. The character of the deviation is determined by the shape of the temperature dependence of the thermal conductivity or electrical conductivity [fitting parameter *α* in Equation (18)]. A breaking of the *I*–*V* characteristic, occurring at some magnitude of the electric field strength, corresponds to the threshold of the thermal instability. The distinctions in behavior of the *I*–*V* characteristics calculated within the framework of various models at high currents are explained by the difference in the maximum tip temperature *T*_max_ corresponding to the limiting emission current. The higher the nanotube’s temperature, the more the deflection of the *I*–*V* characteristic from the Fowler-Nordheim dependence, and the sharper the bending of a tail of the curve.

Figure 7b presents the dependences of the limiting emission current *I*_max_ on the ratio *R*/*λ*, *i.e.*, on the limiting value *T*_max_ of the tip temperature, obtained for five models of calculation of *I*–*V* characteristics. As is seen, the character of the dependences considered is quite weakly sensitive to the accepted assumption about the temperature dependences of transport coefficients. This indicates the universal nature of the instability under consideration, which manifests itself independently of model assumptions.

Another approach to describing thermal phenomena in CNT-based emitters has been developed by the authors of [64]. In accordance with their concept, an emitter is destroyed upon reaching the melting point. The critical parameters of emission (current, voltage, and temperature) were calculated using the following approximation of the temperature dependence of the CNT resistivity: *ρ*(*T*) = *ρ*_o_ (1 − *αT* + *βT*^3/2^) where the fitting parameters *ρ*_o_ = 3.26 × 10^−5^ Ω·m, *α* = 8.5 × 10^−4^ K^−1^, *β* = 9.8 × 10^−6^ K^−3/2^. The thermal conductivity coefficient was taken equal to 100 Wm^−1^ K^−1^ and assumed to be temperature independent. The calculated results imply a nonlinear character of dependences of limiting (critical) temperature, electric field strength, and the emission current density on the nanotube’s length. Thus, the dependence of the critical temperature on CNT length shows a nonmonotone behavior and takes the minimum value at *h* ≈ 19 μm. A further increase in the nanotube’s length is accompanied by a rise in the critical temperature and the critical emission current. This is caused by a lowering of the effectiveness of the heat removal as the nanotube’s length increases.

It should be noted that thermal effects can have a considerable influence on the operation of electron field emitters of any nature. However, the thermal instability phenomenon under consideration is apparently inherent to only CNT-based cathodes. Indeed, in contrast to conventional electron field emitters that have a conical structure, nanotubes represent 1D conducting systems possessing an elongated cylindrical form. There exists a natural limitation on the rate of heat transport through the cross section of a nanotube. As the emission current is enhanced, the heat generation increases, while the rate of heat removal hardly changes and can even decrease in the case of a dropping temperature dependence of the thermal conduction coefficient of the nanotube. This can ultimately result in an unlimited rise in the temperature of the nanotube near its tip and its thermal decomposition.

The influence of thermal effects on the emission properties of CNT-based electron field emitters has been observed in many studies. Thus, the authors of [65], who studied the emission behavior of an individual multiwalled CNT about 0.5 mm in height and about 10 nm in diameter, observed a catastrophic decomposition of the emitter upon exceeding an emission current of 0.2 mA. However, another publication [66] reports a lower value of the maximum reachable emission current by about an order of magnitude. Apparently, the thermal stability of CNT-based emitters is rather sensitive to the type and content of defects in nanotubes, which in turn depends on the procedure and conditions of their production.

Thermal decomposition of CNT-based emitters due to Ohmic heating during emission has been studied in detail in [66]. Based on the analysis of experimental data, the authors concluded that the decomposition of CNTs at comparatively low magnitudes of the applied voltage is due to the mechanical failure of the contact between the nanotube and the substrate, while the main reason for decomposition at high values of the applied voltage is the Ohmic heating of the emitter. Measurements evidenced that the current-voltage characteristic of such an emitter at relatively low currents corresponds to the Fowler-Nordheim dependence, reaching saturation as a result of the increase in the current. Further enhancement of the current is accompanied by the thermal decomposition of the emitter. The saturation current amounts to 920 nA for a nanotube 0.66 mm in length and 5 nm in radius, while irreversible thermal decomposition is observed at a current of about 7.5 mA. Treatment of the current-voltage characteristic represented in the Fowler-Nordheim coordinates permitted the evaluation of the electric field amplification factor *β* = 110 ± 20 and the area of the emitting surface of 3 × 10^−15^ m^2^, supposing the electron work function of 5.1 eV. Whereas the estimated value of the parameter *β* is in reasonable agreement with the approximate relation *β* *≈ h*/*d*, the estimated area of the emitting surface is about 30 times higher than the geometrical cross section of the nanotube.

One more manifestation of the thermal mechanism of nanotube decomposition due to Ohmic heating was found in the experiment [66] performed in a two-probe configuration. First, electron field emission occurring in the above-described configuration was observed upon exceeding the voltage across the gap between the nanotube and anode of 112 V. The current-voltage characteristic at low currents is well described by the Fowler-Nordheim Equation (1) reaching saturation at a voltage of 160 V, and a current of 50 nA. Then, the open end of the nanotube was put into contact with an anode in order to measure the current-voltage characteristic in the two-probe configuration. The thermal decomposition of the nanotube was evidenced at a voltage of 4 V and a current of 20 mA, so that two parts of the nanotube remained attached to the anode and cathode surfaces. The field emission from the fragment of the nanotube remained on the cathode surface was observed at a voltage exceeding 43 V, following the Fowler-Nordheim relation until the emitter was destroyed at a voltage of 108 V and a current of 9 mA.

As the emission current rises, the nanotube temperature increases, which changes not only the character but also the mechanism of emission. Along with electron field emission, which is a result of the tunneling of electrons through the barrier formed at the boundary of a conductor, some contribution to the emission is due to thermionic emission, in which case electrons do not oblige to overcome a potential barrier. The relative contribution from the thermoionic emission rises as the temperature increases, so that the higher the electric field strength, the higher this contribution. The transition of electron field emission to thermionic emission was studied in detail theoretically by the authors of [67], who utilized the tight binding approximation to describe the electronic characteristics of a (12; 0) single-walled nanotube. The calculations showed that the field emission mechanism prevails for *T* < 1000 K within the range of the electric field strength 2 < *E* < 8 V nm^−1^, where the current-voltage characteristic follows the Fowler-Nordheim equation. For *T* > 1000 K, electron field emission prevails as *E* < 6 V nm^−1^, while at higher fields the contribution of the thermionic emission becomes decisive. Therefore, the relative contribution of thermionic emission has a minimum at *E* ≈ 5–6 V nm^−1^. Naturally, the position of this minimum depends on the temperature.

### 2.4. Electrical Field Induced Alignment of CNTs

A CNT possesses very elongated structure, and its orientation is susceptible to the action of the electrical field. The electrical field manifests itself during the growth of CNT, providing its rectilinear structure and preferable orientation. Besides of that the electrical field acting onto nanotubes during the emission promotes the orientation of initially tilted nanotubes which changes the current-voltage emission characteristics. One more effect of the electrical field relates to the contact potential difference between the nanotube and the material of the substrate. This effect makes CNTs to be charged relatively the substrate and distorts the spatial distribution of the electrical field in a vicinity of a nanotube. All these effects call for detailed theoretical and experimental study of influence of the electric field on the alignment of CNTs during their growth and emission. A research of such a kind has been performed and published in [67]. Some results of this work have been presented below.

#### 2.4.1. Growth of an Elongated Structure under the Action of the Electrical Field

Abstracting from a chemical nature of the CNT growth, we consider the initial stage of the growth of an extended structure as a result of mutual attraction of neutral spherical particles under the action of an external electric field [68,69]. Electric field **E** induces a dipole moment in a neutral particle: **D** = *α***E**. Therefore, the interaction potential of two neutral particles at large distances *R* from each other is determined by the values of these induced dipole moments **D**_1_ and **D**_2_ and is expressed by the following relation:

*U* = [**D**_1_**D**_2_ − 3(**D**_1_**n**)**D**_2_**n**)]/ *R*^3^ = −*α*_1_*α*_2_*E*^2^(3 cos^2^*θ* − 1)/ *R*^3^ (19)

Here, **n** is the unit vector directed along the line connecting the centers of the dipoles, *θ* is the angle between vectors **n** and **E**, and the dipole moment of a particle is expressed in terms of its polarizability *α* by Equation (18). It follows from Equation (19) that for small angles 0 < *θ* < arccos(1/3), electrostatic attraction appears between the particles, which results in the growth of an elongated structure. Normal *F_n_* and tangential *F_t_* components of the interaction force between the induced dipoles are given by:

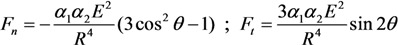
(20)

Since the attractive force is most significant only for small values of angles *θ*, the tangential component of this force can be neglected. In this case, the value of angle *θ* does not change when the particles approach each other, so the time of approach of the particles located at distance *R* from each other at the initial instant is determined from the equation of motion in a viscous medium:

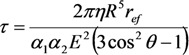
(21)

Here, *η* is the viscosity of the medium and *r*_ef_ is the particle effective radius, which describes viscous drag acting on the particle moving in the medium. Averaging this relation over the initial positions of the particles, one obtains:

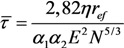
(22)
where *N* is the density of the particles. The polarizability of a spherical conducting particle with radius *r* is estimated as *α* ≈ *r*^3^. Taking into account this estimate and Equation (22) one obtains:

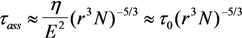
(23)
where *τ*_0_ = *η*/*E*^2^ is the characteristic time of the problem. For the typical conditions of the CNT synthesis by the method of chemical vapor deposition (CVD), *η* ≈ 3 × 10^–5^ Pa s, and *E*≈ 10^3^ V/cm, (*r*^3^*N*) ≈ 10^–7^ to 10^–6^, whence follows *τ*_0_ ≈ 10^–5^ to 10^–4^ s and *τ* ≈ 10^5^–10^6^ s. This time seems to be too long; therefore, the mechanism under consideration is scarcely significant in the situation involved.

One more mechanism of association of the particles includes their diffusion towards each other. The rate constant of this process is defined by the Einstein–Smoluchowski formula:

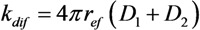
(24)
where *D*_1_ and *D*_2_ are the values of the diffusion coefficients of particles in the gas, which are expressed in terms of the Einstein relation taking into account the Stokes formula:

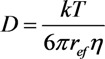
(25)

This leads to the following estimate of the association time, which in this case is independent of the electric field strength:

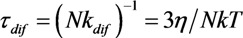
(26)

If a small spherical particle with radius *r* attaches to an extended particle of length *l*, then Equation (26) takes the form:

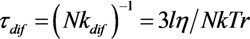
(27)

The ratio of the quantities estimated using Equations (23) and (26) can be written in the form

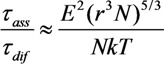
(28)

This expression contains two small parameters: (*r*^3^*N*) ≈ 10^–6^, which is in essence the criterion of an ideal gas, and *E*^2^/*NkT* ≈ 10^–9^, which indicates a small value of the electric field energy per molecule as compared to its thermal energy. Thus, the obtained estimates show that under typical conditions of the CNT synthesis by the CVD method, the basic mechanism of the association of particles at the initial stage of growth is the mechanism including the particle diffusion, which means that the electric field does not produce any appreciable effect on the particle growth. According to Equation (26), the characteristic time of the particle association is estimated as *τ*_dif_ = 3*η*/*NkT* ≈ 10^–8^ s. This estimate corresponds to the initial stage of formation of the extended structure.

The electric field influence on the growth of the structure increases with its length. This is due to the effect of the electric field enhancement near the tip of the cylindrical tube. The angular dependence of the longitudinal component of the electric field strength contributes to the preferred attachment of the particles incident from the end side of the tube. This causes its growth in the longitudinal direction. Let us consider the growth of an extended structure taking into account the effect of the electric field enhancement. The electric field applied to the extended conducting cylinder induces a dipole moment, which is given by the following relation:

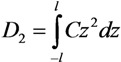
(29)
where the direction of the *z* axis coincides with the cylinder axis and *σ*(*z*) = *Cz* is the specific electric charge per unit length of the cylinder. Integrating Equation (29), we obtain the following expression: *C* = 3**D**_2_/(2*l*^3^) = 3*α*_||_*E*/(2*l*^3^), where *α*_||_ = –*l*^3^/[3ln(*l*/*r*)] is the longitudinal component of the polarizability of the cylinder and *r* is its radius. The potential of interaction of a spherical particle, which has dipole moment **D**_1_ induced by the electric field, and the extended cylinder with dipole moment **D**_2_ can be written in the following form:

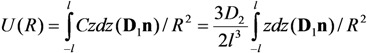
(30)

Here, **n** is the unit vector directed along vector *R*. Integration along the cylinder axis with the use of polar coordinates *z* and *ρ* (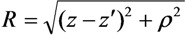, *z*' is the coordinate along the cylinder axis) results in the following expression:

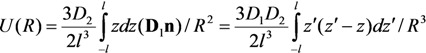
(31)

In the case of an extended cylinder with *l >>R*, the region near the tip of the nanotube makes the main contribution to integral [Equation (31)]. This permits us to replace *z*' by *l* which result in the following relation:

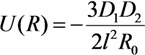
(32)
where 
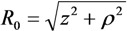
 is the distance from the spherical particle to the cylinder tip. Equation (32) is valid in the region *z* > *l*. This equation yields the formula connecting the electric field strength and the force exerted by the conducting cylindrical tube on the spherical particle in the field:

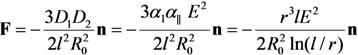
(33)

Assuming that the position of the extended structure is fixed and using the Stokes formula:

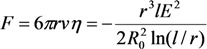
(34)
to describe the motion of the spherical particle under the action of the above force, we obtain the equation of motion for the particle:

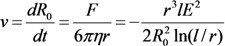
(35)

The solution of this equation leads to the following expression for the time, during which the particle reaches the surface of the elongated structure:

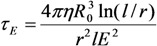
(36)

By averaging this equation taking into account the distribution of distances *R*_0_ between the nearest particle and the CNT tip, we obtain:

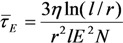
(37)
where *N* is the concentration of the particles. This expression is valid if *Nl*^3^ >> 1. As it follows from Equation (37), the characteristic time for attachment of a small particle to an elongated structure of length *l* is inversely proportional to the value of this parameter, which is due to the effect of the electric field enhancement in the vicinity of the CNT tip. However, the growth rate of the extended structure is determined by the initial (slowest) stage of the growth evolution. The growth duration of the structure of length *L* can be obtained by time averaging of Equation (37) taking into account the change in its length:

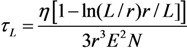
(38)

It can be seen that the growth duration of an elongated structure with *L >>* *r* depends critically on size *r* of the particles and is weakly sensible to length *L* of the tube. The time of growth becomes shorter upon an increase in electric field *E* and concentration *N* of the particles.

The typical value of the electric field strength, which is applied to the substrate to provide the growth of vertically aligned CNTs during the CVD synthesis, is ~10^2^–10^3^ V/cm [70,71,72]. Substituting these values together with the CNT-typical quantities *r* ≈ 10^–8^ cm and *N* ≈ 10^–18^ cm^–3^, we can estimate the time of growth of an extended cylindrical structure at *L* ≈ 10^2^–10^3^ s, which agrees well with the actual growth conditions [70,71,72].

The above estimates make it possible to understand the physical reason for the advantage of the growth of the extended structure over the aggregation of small particles in a cluster. In the vicinity of the tip of the extended structure with the aspect ratio *a* = *l*/*r*, the effect of the electric field enhancement takes place, due to which the local value of the field is approximately *a* times higher than the average field strength in the gap. Thus, the ratio of the average time [Equation (37)] of the particle attachment to an extended structure to the characteristic time of aggregation of small particles into the cluster defined by Equation (23) is estimated by the following relation:

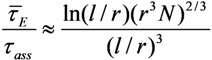
(39)

This relation is independent of both the electric field strength and the viscosity of the medium. It contains two small parameters, which determine the preferred growth of an elongated structure as compared to aggregation of small particles into a cluster.

#### 2.4.2. Alignment of CNTs under the Action of the Electrical Field

**Figure 8:**
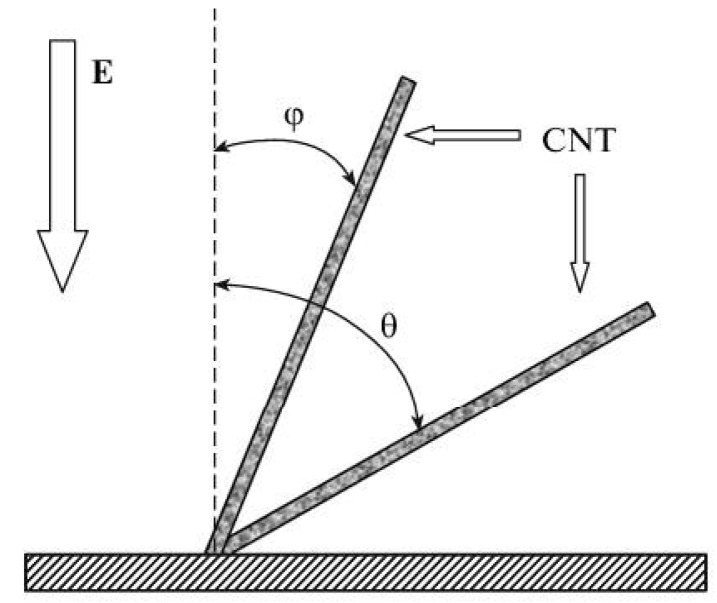
Configuration of the nanotube inclined relative to the substrate and aligned under the action of the electric field; *θ* is the initial angle of inclination of the nanotube and *φ* is the resultant angle of inclination.

The nanotubes constituting an electron field emitter are usually not aligned strictly vertically. In this case, the angle of nanotube orientation relative to the substrate depends on the strength of the external electric field directed perpendicularly to the plane of the substrate. Thus, a problem arises on the dependence of the CNT orientation angle relative to the substrate on the electric field strength. This problem will be analyzed below for the configuration shown in Figure 8.

The nanotube initially aligned at angle *θ* relative to the substrate is partially straightened as a result of the action of the electric field so that the orientation angle becomes *φ*. The value of this angle can be determined from the mechanical balance of the forces acting on the nanotube. Electric field *E* induces polarization *P* of charges in the conducting nanotube, equal to *P* = *α*_||_*E*cos*φ*. Here, *α*_||_ is the longitudinal polarizability of the tube. The interaction between the electric dipole formed in such a way and the electric field generates a torsion moment *M* [68,73], which is expressed by the following relation:

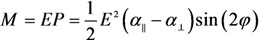
(40)

According to numerical computations [73], the following relations are valid for the longitudinal and transverse polarizability of the CNTs:

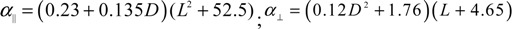
(41)
where *D* and *L* are the diameter and length of the nanotube (Å), respectively. As can be seen, the longitudinal polarizability is approximately *L*/*D* times higher than the transverse polarizability; therefore, for the nanotubes with large aspect ratio *L*/*D>>* 1, the transverse polarizability is negligibly small as compared to the longitudinal polarizability.

The bending deformation of a nanotube under the action of the electric field is balanced by the elastic force; its value is described by the mechanical elastic constant defined as [73]:

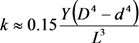
(42)
where *Y* is the Young modulus, and *D* and *d* are the outer and inner CNT diameters, respectively. Usually, *D*^4^ − *d*^4^ ≈ *D*^4^ for a multilayer nanotube, so that we can approximately write:

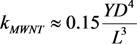
(43)

For a single-layer CNT, *D*^4^ − *d*^4^ ≈ 4*δD*^3^, where *δ* ≈ 3.4 Å is the effective thickness of the nanotube wall. The angle of CNT bending under the action of the electric field is determined from the equation of mechanical balance between the bending moment and the moment of elastic forces:

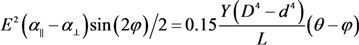
(44)
where angle *θ* − *φ* is the CNT bending angle. Equation (44) permits the estimation of the value of electric field strength *E_c_*, which compensates the initial bending of the nanotube. Taking into account the condition *φ* << 1, we can write this estimate in the following form:

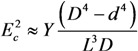
(45)

In the case of a multilayer CNT, the Equation (45) is reduced to the following relation:

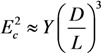
(46)
while in the case of a single-layer CNT, one can write:

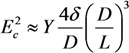
(47)

By specifying the parameters *Y* = 200 GPa, 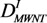 = 5 nm, 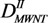 = 20 nm, and *L* = 5 μm of multilayer CNTs, we can estimate the value of the electric field strength required to ensure the vertical alignment of CNTs: 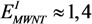 V/μm and 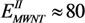 V/μm. For a single-layer CNT of the same length (*Y* = 1000 GPa and DSWNT = 1 nm), estimate Equation (47) yields *E*_SWNT_ ≈ 0.3 V/μm. Thus, the above estimates show the possibility of the CNT vertical alignment as a result of application of the external electric field. In this case, the extent to which the CNT alignment is affected by the electric field is determined by the aspect ratio of the nanotube, as well as by its mechanical characteristics, which are determined by the value of the Young modulus.

**Figure 9:**
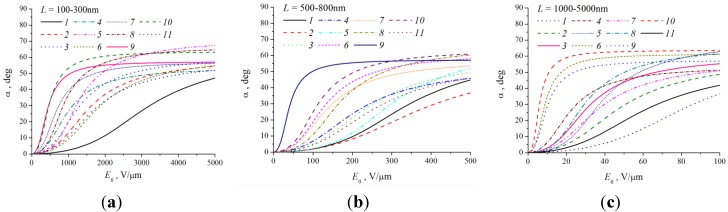
Dependences of angle of inclination *α* = *θ* – *φ* on the local value of the electric field strength, computed for the CNTs with parameters given in the table. The values of *L*, nm, are as follows: (**a**) 100–300, (**b**) 500–800, and (**c**) 10^3^–5 × 10^3^.

Figure 9 shows the results of computations of the dependences of the nanotube angle of bending under the action of the electric field on the local value of field strength *E*, calculated on the basis of the mechanical balance Equation (44). The computations were performed for CNTs of different lengths, divided into three groups. The CNT parameters chosen at random with the help of the random number generator from a certain range of values are specified in the Table 2. It can be seen that the saturation region of the computed dependences is determined by the geometrical parameters of the nanotube, as well as by its mechanical properties (the Young modulus).

**Table 2:** Parameters of the nanotubes for which the computations represented in Figure 9 are performed.

No.	1	2	3	4	5	6	7	8	9	10	11
*D* (nm)	49.3	45.8	46.8	27.9	38.7	19.5	22.2	27.1	10.4	15.5	42.7
*θ* (°)	66.3	59.1	60.2	53.6	71.1	61.4	57.1	66.3	57.4	63.6	57.4
*Y* (GPa)	36.9	45.2	18.9	34.7	19.8	44.2	47.4	30.5	49.7	43.0	25.6
*L* (μm) (0.1–0.3)	0.183	0.286	0.252	0.214	0.214	0.109	0.237	0.216	0.153	0.206	0.210
*L* (μm) (0.5–0.8)	0.754	0.695	0.730	0.573	0.524	0.602	0.674	0.633	0.732	0.524	0.702
*L* (μm) (1–5)	1.88	2.76	2.80	2.57	2.072	3.66	1.62	1.98	1.87	3.70	1.79

### 2.5. Degradation of a CNT-Based Emitter

The main issue, determining the applicability of CNTs as emitters in electron field cathode relates to the lifetime of these emitters which is governed by the degradation of emitters during the operation. The following three degradation mechanisms of CNT-based electron field emitters are known: contact failure, thermal decomposition, and ion sputtering [74]. The degradation mechanism related to contact failure has no universal character and manifests itself when insufficient attention is paid to the contact formation. This problem can be solved by using a buffer layer between a substrate and nanotubes. The basic mechanism of the degradation of CNT-based emitters that restricts the emission current is the thermal decomposition of nanotubes because of Joule heat. The detailed analysis of this mechanism performed above (see Section 2.2.) demonstrates that the effect has a character of thermal instability and manifests itself at a certain critical emission current, when the thermal balance between the Ohmic heating of CNTs during emission and the heat removal via thermal conduction is violated. The threshold character of this effect can be used to avoid the thermal decomposition of CNTs by operating at voltages (currents) below the critical value. In contrast to this effect, the ion sputtering mechanism manifests itself at any applied voltage and emission current [74]; it should be considered separately, as it is done below. The ions forming during the ionization of residual gas molecules by an electron impact are accelerated in an electric field and bombard the emitter surface, which leads to its ion sputtering. The energy spectrum of the ions inducing the sputtering of the cathode material is determined by the spatial distribution of the electric potential in an interelectrode gap. The dependence of the sputtering probability from the ion mass and energy is expressed by well-known classical relationships supported by numerous experiments.

One should note that the initial approach to the description of the above-mentioned mechanism of the sputtering has been developed in [74,75]. This approach is based on a simplifying supposition neglecting the thermal motion of residual gas molecules whose ionization is a source of formation of ions bombarding the cathode. Due to this proposition the ion trajectory coincides (and oppositely directed) with that of the electron promoted the ionization. Therewith all the ions formed move along the force lines of the electrical field and participate in the bombardment of the emitter (nanotube). However estimates performed in [74,75] demonstrate that the inclusion of the initial motion of the residual gas molecules results in the only some fraction of ions reaches the nanotube’s surface, while the rest of ions due to the initial motion in radial direction fall on the substrate missing the emitter and do not promoting its degradation. This effect is studied quantitatively below.

#### 2.5.1. The Trajectory of Ions

**Figure 10:**
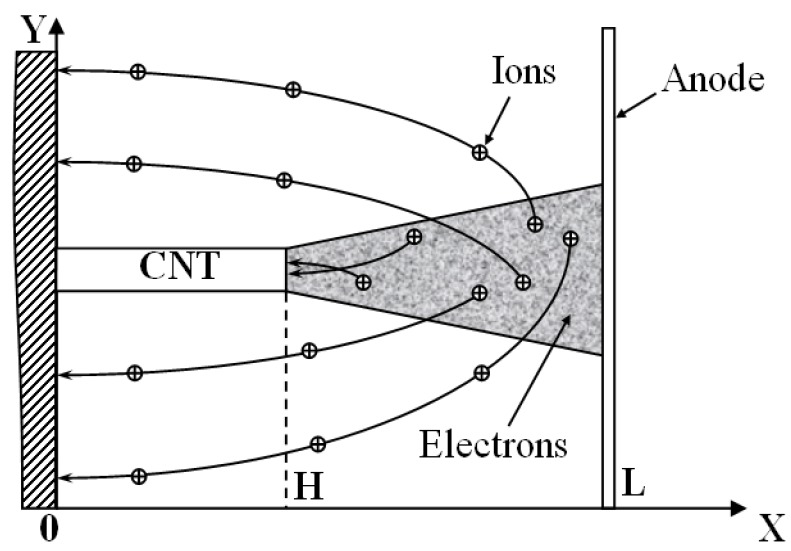
The model configuration of the emitter under consideration.

Figure 10 presents the model configuration for which the sputtering rate will be calculated. The nanotube of *Н* in height and *R* << *H* in radius is oriented vertically to a ground conducting substrate. The action of a longitudinal electric field promotes the electron field emission from the nanotube’s tip. The ions formed as a result of the electron impact ionization of the residual gas molecules bombard the surface of the nanotube. While the temperature of the vacuum camera (~0.1 eV) is as low as many orders of magnitude comparing to the characteristic ion energy (~10^2^–10^3^ eV), one should take into account the initial motion of the residual gas molecules from which ions form as a result of electron impact. This is seen from the estimation of the characteristic size of the ion spot on the target surface: 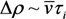. Here *v* ~ (2*T*/*M*)^1/2^ ~ 5 × 10^4^ cm/s is the characteristic thermal velocity of the residual gas molecules; *τ*_i_ ~ (*ML*/2*eE*)^1/2^ is the characteristic time for which an ion formed within the inter-electrode space and moving towards the cathode surface under the action of the electrical field reaches this surface; *L* is the inter-electrode distance; *М* is the mass of a residual gas molecule. Inserting *М* ≈ 5 × 10^−23^ g (for air molecules); *L* = 10^−2^ cm; *Е* = 10^4^ V/cm, one obtain the estimation *τ*_i_ ~ 0.4 × 10^−8^ s. Therefore the characteristic displacement of an ion in respect to the position of the CNT, from which the relevant electron was emitted even for very short inter-electrode gaps *L* = 10^−2^ cm accounts 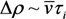 ~ 10^−4^ cm, which exceeds the characteristic radius of a nanotube *R* ~ 10^−6^–10^−5^ cm as much as an order of magnitude. This estimation demonstrates that a main fraction of ions reaching the cathode surface does not fall onto the nanotube and therefore does not promote its degradation. This determines the necessity of the establishing the task on the degradation of a CNT as a result of the ion sputtering with taking into account the initial thermal motion of the residual gas molecules.

It is supposed that a vertically oriented nanotube inserted into a vacuum gap of *L* in length and experienced to the action of the electrical field, emits a thin cylindrical electron beam of radius *R*. Collisions of the with the residual gas molecules promotes the formation of ions moving towards the cathode surface. Assuming that the ion is formed from molecules being in rest one obtain that the ion trajectory coincides (and oppositely directed) to that of the electron whose collision with molecule resulted in the ion formation. Discarding this assumption and taking into account the initial thermal motion of the residual gas molecules results in some displacement of the position of the point of ion bombardment relating to the positioning of the nanotube.

Analyzing the trajectory of ions formed as a result of the electron-molecule impact one can neglect the longitudinal component of the initial thermal velocity of molecules. This follows from the comparison of the characteristic time *τ*_i_ for which the ion reaches the cathode surface with the time *τ*_Е_ for which ion loses its initial velocity directed against the electrical field. The estimation *τ*_Е_ = 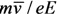 ~ 10^−10^ s shows that the latter is more than an order of magnitude lesser than the above-estimated value of *τ*_i_. This means that taking into account the initial longitudinal motion of molecules results in a minor (within several percent) changing the time of reaching the cathode surface and therefore the displacement of the ion Δ*ρ*. Neglecting this longitudinal component the distribution of the ion over the cathode surface is represented as follows:

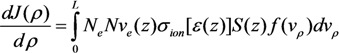
(48)

Here *σ*_ion_[*ε_e_*(*z*)] is the electron impact ionization cross section for the residual gas molecule at the collision energy *ε_e_* that is determined by the potential of the point *z* relating to the ground cathode surface:

*ε*(*z*) = *eU*(*z*) (49)

*N* is the number density of the residual gas molecules which is constant over the inter-electrode space, *N*_e_(*z*) is the local value of the electron number density that is expressed through the electron current density as *j*_e_ = *N*_e_(*z*)*v*_e_(*z*); *S*(*z*) is the cross section of the electron beam which is changed due to its angular divergence; *v*_e_(*z*) is the velocity of an emitted electron in the point *z* that is determined by the electrical potential in this point: *v*_e_(*z*) = [*eU*(*z*)/2*m*]^1/2^); 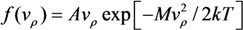 is the equilibrium (maxwellian) distribution function of the residual gas molecules over the radial velocities, *А* = *M*/*kT* is the normalization constant, *e*, *m* is the electron charge and mass, correspondingly, *М* is the mass of the residual gas molecule, *Т* is the temperature. The Equation (48) is simplified by the use of a natural equation *I_e_* = *j*_e_*S* (*I*_e_ is the emission current) which is valid for homogeneous distribution of the electron number density over the cross section of the beam. Violation of this assumption does not change the qualitative results of the present research. Note that the Equation (48) presents the number of ions bombarding per second the surface (*ρ*, *ρ* + *dρ*), so that the dimensionality of *J* is cm^−1^s^−1^.

The initial radial thermal velocity of an ion *v*_ρ_ inter-relates with the radial displacement of the point where the ion reaches the cathode surface in respect to the point (*ρ* = 0, *z* = 0) through the following expression:

*v*_ρ_*τ*_i_(*z*) = *ρ* (50)

where *τ*_i_(*z*) = (*Mz*/2*eE*)^1/2^ is the time for which the ion formed in the point *z* reaches the cathode surface.

The integration of Equation (48) should be performed for some specific distribution of the electric potential over the gap. The simplest form of such a distribution obeying the conditions *U*(*z* = 0) = 0; *U*(*z* = *L*) = *U* is the linear function:

*U*(*z*) = *Uz/L* (51)

which is inherent to a flat capacitor. The Equation (51) is violated in a vicinity of the nanotube’s tip where the effect of the electrical field enhancement occurs, how it is easy to prove that the electron energy in this region is still insufficient for ionization of the residual gas molecules, therefore this region does not contribute into the integral [Equation (48)].

Equation (48) with taking into account the above-given relations [Equations (49)–(51)] has the following form:

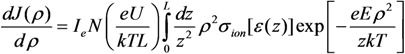
(52)

**Figure 11:**
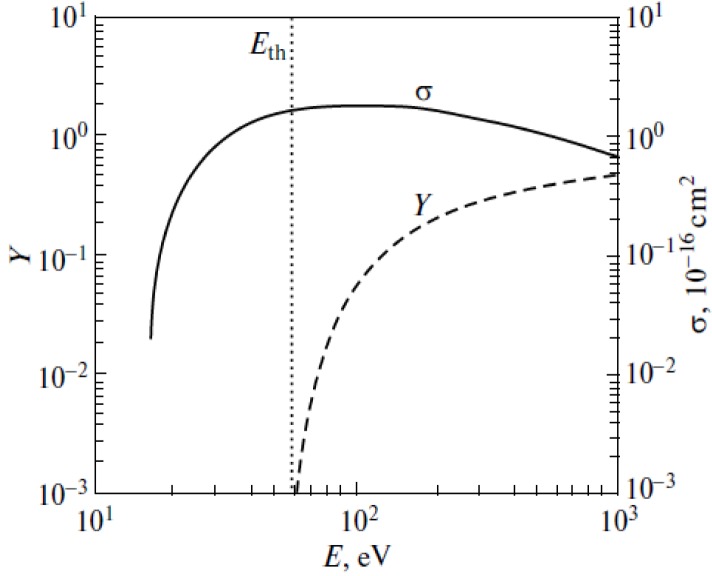
Energy dependences of the ionization cross section of a nitrogen molecule by an electron impact σ [76] and ion sputtering coefficient *Y* [78] for the bombardment of the graphite surface by molecular nitrogen ions. *E*_th_ is the threshold sputtering energy.

The result of integration presents the distribution of the electron current over the cathode surface. The energy dependence of the electron impact ionization cross section for nitrogen is presented on Figure 11 [76,77]. The high energy tail of this dependence has been obtained by extrapolation of the experimental curve [76] with the use of the Born approximation. Figure 11 contains also the energy dependence of the ion sputtering coefficient that will be specified below.

**Figure 12:**
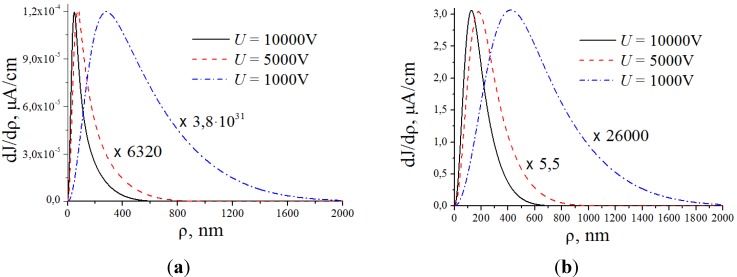
Distribution of the ion current *J* over the target surface *ρ* calculated at various magnitudes of the applied voltage *U*. (**a**) The main calculation parameters: nanotube’s radius *R* = 5 nm, nanotube’s height *H* = 1 μm, residual gas pressure *P* = 10^−4^ torr, temperature *T* = 1000 K, inter-electrode distance *L* = 100 μm. The electrical field strength near the nanotube’s tip *Е* = 10 V/nm, 5 V/nm and 1 V/nm correspondingly. The curves for *V* = 5000 V and 1000 V are given in an enlarged scale. (**b**) The main calculation parameters: *R* = 5 nm, *H* = 10 μm, residual gas pressure *P* = 10^−4^ torr, temperature *T* = 1000 K, inter-electrode distance *L* = 100 μm. *Е* = 10 V/nm, 5 V/nm and 1 V/nm correspondingly. The curves for *V* = 5000 V and 1000 V are given in an enlarged scale.

Figure 12 shows several typical distributions of the ion current over the cathode surface calculated with the use of the data given on Figure 11 on the basis of the Equation (52) for various values of inter-electrode distance *L*, average electrical field strength *E* = *U*/*L* and the aspect ratio of the nanotube *α* = *H*/2*R*. There was assumed that the main sort of the residual gas is air. There was assumed that the inter-relation between the emission current and the electrical field strength near the nanotube’s tip obeys the Fowler-Nordheim relation (1) with taking into account the field enhancement effect. As is seen the part of the electron current reaching the nanotube’s surface decreases as the inter-electrode distance increases and the applied voltage lowers. This effect is caused by the role of the initial thermal motion of the residual gas molecules.

#### 2.5.2. The Degradation Rate and the Effective Lifetime of an Emitter

The rate of degradation of the material of an emitter due to the ion sputtering is defined as the mass of the emitter lost in the unit of time and is calculated through the following relation:

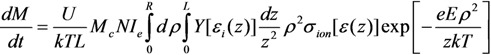
(53)
where *M*_CNT_ is the mass of the nanotube depending on its radius and height (thus for *R* = 5 nm and *H* = 10 μm *M*_CNT_ = 2.4 × 10^−16^ g); *М*_с_ is the mass of carbon atom; *Y*(*ε*_i_) is the ion sputtering coefficient determined as the average number of atoms escaping from the emitter’s surface as a result of its bombardment by an ion having the energy *ε*_i_. This coefficient is determined by the type of the ion, the surface material and the ion energy *ε*_i_ near the cathode surface. The characteristic lifetime of an emitter *t* was calculated with the use of Equation (53) through the following relation [74,75]:

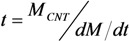
(54)

The energy dependence of the ion sputtering coefficient *Y*(*ε*_i_) was determined on the basis of the classical approach [78], in accordance with which the sputtering is resulted from the elastic scattering of an ion when the energy transferred exceeds the atom binding energy. There was assumed that the binding energy of a carbon atom in a CNT lattice is similar to that in crystalline graphite. The energy dependence of the ion sputtering coefficient for nitrogen molecular ion is shown on Figure 11 by the broken line. Since the energy dependences of the electron impact ionization cross section and the ion sputtering coefficient for nitrogen and oxygen coincide practically, the specific calculations were performed for nitrogen, assuming applicability of the calculation data also for air as the residual gas.

**Figure 13:**
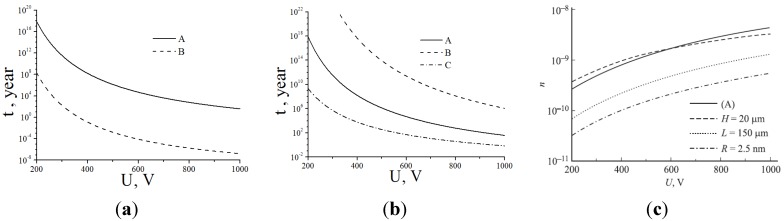
The results of calculations: (**a**) Comparison of the dependences calculated with (A) and without (B) taking into account the initial thermal motion of residual gas molecules. *R* = 5 nm, *H* = 10 µm, *L* = 100 µm. (**b**) The dependences *t*(*U*) calculated taking into account the initial thermal motion of residual gas molecules for various parameters of the system: (A) *L* = 100 µm, *H* = 10 µm, *R* = 5 nm; (B): *L* = 200 µm, *H* = 10 µm, *R* = 5 nm; (C): *L* = 100 µm, *H* = 20 µm, *R* = 5 nm. All the calculations were performed for *P* = 10^−4^ torr, *T* = 1000 K. (**c**) Ratio of the degradation rate calculated with and without taking into account the initial thermal motion of residual gas molecules. Calculation parameters: (A) nanotube radius *R* = 5 nm; height *H* = 10 μm; residual gas pressure *P* = 10^−4^ torr; temperature *T* = 1000 K; interelectrode distance *L* = 100 µm. In the rest calculations one parameter has been changed as shown on the relevant curves.

**Figure 14:**
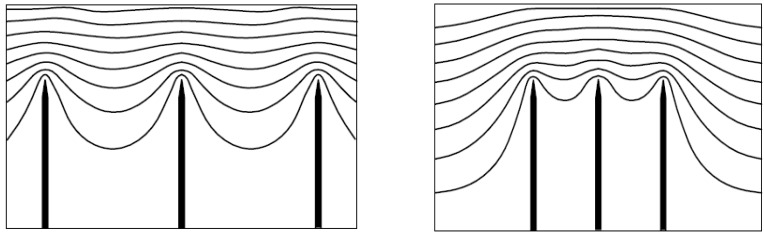
Spatial distribution of the electric potential in a vicinity of three CNTs calculated for various inter-tube distances (arbitrary units) [28]. The closer nanotubes the lower electrical field enhancement factor.

The characteristic lifetime of the emitter calculated on the basis of Equations (53) and (54) for various magnitudes of the electrical field strength, inter-electrode spacing and the nanotube’s aspect ratio is presented on Figure 13. Comparison of the data obtained with and without taking into account the initial thermal motion of residual gas molecules indicates a notable role of the thermal motion of molecules in decreasing of the sputtering degradation of the CNHT-based electron field emitters. An abrupt dependence of the lifetime of the applied voltage relates to a sharp shape of the Fowler-Nordheim Equation (1). The role of the thermal motion of the residual gas molecules in the degradation mechanism under consideration can be seen also from Figure 14 presenting the ratio of the degradation rates calculated with and without taking into account the thermal motion of residual gas molecules. The calculation was performed by the formula:

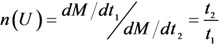
(55)

One should keep in mind that the above-introduced definition of the emitter’s lifetime [Equation (54)] has a conventional meaning and should not be taken verbatim. This relates to a decrease in the nanotube length during the degradation. This decrease is accompanied by a decrease in the electric field gain. As a result, the emission current decreases in time at a fixed applied voltage. As follows from the Fowler–Nordheim relationship, the electric field gain enters into the exponent of a sharply changing exponential function; therefore, a small change in the nanotube length during degradation is accompanied by a substantial decrease in the emission current at a fixed applied voltage. This effect of a change in the CNT geometry during emission has not been taken into account during the calculations; therefore, the calculated emitter lifetimes are assumed to be related to the situation where an emission current is constant. However, the applied voltage is not constant in this case. Nevertheless, the calculation results demonstrate that the value of integral [Equation (52)] in the main voltage range remains almost unchanged, so that the effect of an applied voltage on the emitter lifetime manifests itself through a sharply changing *I*–*V* characteristic, which corresponds to the Fowler–Nordheim relationship. Thus, although the determination of the CNT lifetime has a formal meaning, this determination is thought to be related to the situation where an emission current is fixed.

The above-presented calculation results relate to an individual nanotube emitting electrons. An electron field cathode contains an array of such nanotubes, and, as it will be explained below, the optimal inter-tube distance lies within the range between 0.3*Н* and *Н* (*Н* is the height of the nanotube), depending on the applied voltage. Since the characteristic height of nanotubes used in electron field emitters ranges between 1 and 10 μm, the optimum inter-tube distance is varied from a fraction of the micrometer to few micrometers. Comparing this value with the distance characterizing the spread of the ion current in a nanotube’s vicinity Δ*ρ* ~ 0.1–1 μm (see Figure 12) one concludes that in an optimal regime the ions formed as a result of ionization by the electrons emitted by a specific nanotube can contribute into the degradation of only this nanotube and do not reach practically neighboring nanotubes. Therefore the above-given results obtained for the degradation rate of an individual nanotube are valid only for a cathode of the optimal geometry containing an array of such nanotubes.

The calculation results demonstrate a high sensitivity of the degradation rate of CNT-based electron field emitters to the applied voltage. This sensitivity is caused by a sharp shape of the *I*–*V* characteristic of the emitter. Another factor affecting the degradation rate is the residual gas pressure. The effect of the kind of residual gas is moderate: different atmospheric air components (N_2_, O_2_) exert approximately the same effect on the degradation rate. The possible difference in the degradation rates for gases of different kinds, which is related to the difference between the electron impact-induced ionization cross sections and the ion sputtering coefficients, was estimated to be 2–3.

### 2.6. CNT-Based Emitters of Alternative Structure

Along with SWNTs and MWNTs, other CNT-based structures were used as electron field emitters. Thus the authors [79] describe a point emitter fabricated from a composite of CNT and WO_3_. An isolated structure emitter presents a vertically aligned cylindrical pillar of 2 mm long and 20 μm in diameter that is able to deliver an electron emission current up to 10 mA. Such an emitter possesses an elongated structure and good electric conductivity and can be considered as an enlarged prototype of an individual nanotube. One more modification of a CNT-based emitter has been reported by the authors [80] who fabricated a fiber of ~100 μm in diameter and 6 mm in length using the standard procedure of spinning single walled CNTs held together by van der Waals forces. Such a fiber demonstrates good emission characteristics (current densities up to 13 A/cm^2^, current time stability up to 30 h and low turn on and threshold fields 0.12 V/μm and 0.21 V/μm, providing the emission current of 1 nA and 1 μA, respectively) and permits quite easy manipulation due to its macroscopic size.

Rectangular blocks of 50, 100 and 140 μm in width and 200, 150 and 300 μm in height and cylindrical blocks of 20 μm in diameter and 5 μm in height consisted of entangled vertically aligned arrays of multiwall CNTs were used by the authors [81] as individual emitters. Such emitters demonstrate quite stable emission with the maximum current ~300 μA (for rectangular blocks) and ~40 μA (for cylindrical blocks) which corresponds to the average current density of about 3 A/cm^2^. Dense package of the individual nanotubes in the blocks hinders the heat removal, which promotes the occurrence of thermal instability at rather low emission currents comparing to the case of isolated nanotubes considered above.

The theoretical description of the emission characteristics of the above-mentioned macroscopic emission configurations can be performed within the frame of the approaches that have been formulated in preceding Sections of the present article. An emitter can be considered as a large size cylindrical tube. In doing so one can find all the above-described specific peculiarities of the emitter such as interconnection between the geometry of the emitter and field amplification factor, dependence of the field amplification factor on the tilting angle of the emitter, influence of the electrical field onto its orientation, and thermal instability phenomena.

## 3. Emission Properties of a CNT Array

The emission characteristics of a CNT array can differ from those inherent to an individual nanotube. There are several physical reasons causing such a difference. First of all, the above-considered effect of the electrical field enhancement depends on the inter-tube distance. As the inter-tube distance approaches to zero, the enhancement effect decreases due to the screening of the electrical field by neighboring nanotubes. This effect determines the optimal inter-tube distance providing the maximum emission current density for the array. The second factor influencing on the emission characteristics of a CNT array is the natural statistical spread in geometry of individual nanotubes. Due to a sharp exponential dependence of the emission current of an individual nanotube on its aspect ratio even a minor difference in the aspect ratio of various nanotubes results in rather high spread in their emission current. Therefore the main contribution into the emission current is due to the most protruded nanotubes having the maximum value of the aspect ratio. One more effect relates to the electrical field-induced orientation of nanotubes. As it was shown above, the electrical field enhancement factor of a tilted nanotube depends notably on the inclination angle [see Equation (9)]. Applying the electrical field changes the inclination angle, therefore the electrical field enhancement factor of a tilted nanotube depends on the magnitude of the electrical field strength. Besides of that carbon nanotubes situated on a conductive substrate possess the intrinsic electrical field caused by the contact potential difference between the nanotube and the material of the substrate. The magnitude of this field is of an order of 1 V/μm which is quite sufficient for influence on CNT alignment. The action of both external and intrinsic electrical field affects the emission characteristics of a CNT array promoting a deviation of those from the classical Fowler-Nordheim Equation (1). The above-listed physical features of CNT-based emission arrays will be considered below in more detail.

### 3.1. Screening Effects

As it was noted above, the effect of the electrical field enhancement depends on the average inter-tube distance. This effect diminishes as the inter-tube distance decreases, which can be seen from Figure 14, presenting the spatial distributions of the electric potential in a vicinity of three CNTs calculated for various inter-tube distances (compare with Figure 2).

One can single out several types of arrangements of nanotubes in an array. The character of the emission from a rarefied homogeneous array of vertically aligned nanotubes is similar to that occurring in an individual CNT. This means that the distribution of the electric potential in the vicinity of the tip of each individual nanotube in the array does not experience the disturbing action of neighboring nanotubes, so that the total emission current is the result of the summation of the contributions from all the individual nanotubes. In the case of a homogeneous array of closely packed vertically aligned nanotubes, the electric potential formed in the vicinity of the tip of each nanotube is screened by neighboring nanotubes, so that the electric field strength depends notably on the density of nanotubes in the array. This leads to a nonmonotone dependence of the emission current density on the density of nanotubes in the array. This dependence can be evaluated on the basis of the solution of the 3D Laplace equation for arrays of various densities. The third type of the arrangement of CNTs in the array refers to a disordered film of entangled nanotubes of various geometries. Such a type of emitter is rather complicated for analysis. It should be noted that the emission properties of such a film are determined mainly by the nanotubes protruding from the array. The number of such emitters is relatively small and the character of their emission is rather unstable and nonreproducible, so that this kind of emitters is characterized by a relatively low level of emission current density, which is determined by the number and the structure of the most notably protruding nanotubes. Therefore, one should believe that the optimum arrangement of nanotubes corresponds to an array of vertically aligned emitters with moderate number density, for which the screening effect has relatively weak influence.

**Figure 15:**
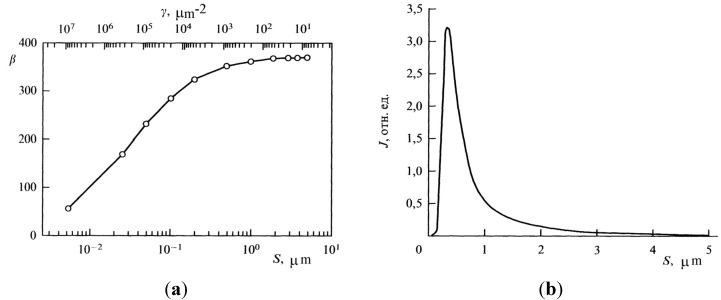
Results of the solution of the 3D Laplace equation for an array of similar vertically aligned nanotubes 1 nm in diameter and 1 μm in height having a flat cap [29]: (**a**) dependence of the electric field amplification factor of a nanotube involved in the array on the average intertube distance *S* and the surface density *γ* of nanotubes; (**b**) dependence of the emission current density of the CNT array on *S*, calculated taking into account the screening effect.

The dependence of the emission current density on the intertube distance in the array was first determined by the authors of [28] (see also [46,47]), who calculated the electric potential for an array of vertically aligned nanotubes as a function of the intertube distance using the 3D Laplace equation. In doing so, the authors established the dependence of the emission current density from the average intertube distance and found the optimal value of this parameter, which turned out to be close to double the height of the nanotube. This result differs somewhat from the conclusion obtained in [29] on the basis of solution of the Laplace equation for a 2D array of similar vertically aligned nanotubes 1 nm in diameter and 1 mm in height having a flat cap. The results of these calculations presented in Figure 15 imply that the optimum value of the average intertube distance providing the maximum emission current density is about half the nanotube’s height.

Subsequent studies [82,83,84] have shown that the quantitative difference in the results of the cited in [28,29] is due to the sensitivity of the form of dependence of the emission current density on the intertube distance in the array to the magnitude of the applied voltage. This relates to the nonlinear character of the Fowler-Nordheim Equation (1) interconnecting the emission current and the voltage across the gap. Therefore, the form of the mentioned dependence is determined by the applied voltage. The value of this parameter is not usually indicated in publications, which can result in misunderstandings.

### 3.2. Statistical Spread of CNT Parameters

As follows from the above analysis, the emission characteristics of CNTs are very sensitive to their individual parameters, like the height, diameter, electron work function and so on. The natural spread in the values of these parameters caused by both the synthesis conditions and changes in the course of the operation promotes notable deviations of the current-voltage characteristics of a CNT array from the Fowler-Nordheim Equation (1) inherent to an individual emitter. A simple approach illustrating the influence of the statistical spread of CNT parameters upon the current-voltage characteristics of a cathode has been developed in [31] (see also [85] containing the similar ideas). There it was supposed that the statistical spread of the electric field enhancement factor *β* of individual nanotubes is described by a normal distribution. The current-voltage characteristic of an individual nanotube obeys the Fowler-Nordheim Equation (1). Due to the statistical spread of the parameter *β*, the magnitude of the electric field strength in the vicinity of the nanotube's tip also has a spread which promotes a distinction of the current-voltage characteristic of the cathode from the Fowler-Nordheim Equation (1). This distinction can be described through a simple model based on the normal distribution of the parameter *β*:

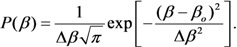
(56)

Here *P*(*β*) is the probability density of the of a specific value *β*; *β*_o_ is the average magnitude of this parameter; Δ*β* is the variance. The emission current density is represented as a result of the statistical averaging the current-voltage characteristic [Equation (1)] with taking account the probability distribution [Equation (56)]:

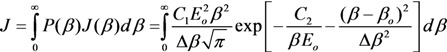
(57)

This integral is easily calculated analytically at conditions:

Δ*β* << *β*_o_;   C_2_/*E*_o_*β*_o_>>1 (58)

First, in this case the low integration limit can be extended down to −∞. Second, the smooth pre-exponent dependence under the integral sign can be neglected comparing to the sharply changing exponent. Third, the first item under the exponent sign can be represented by the following expansion:

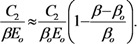
(59)

Calculation of Equation (58) with taking account the above-listed simplifications results in the following relation:

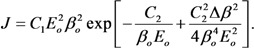
(60)

This expression can be considered as a generalized Fowler-Nordheim equation. This relation contains two factors, one of which corresponds to the classical Fowler-Nordheim Equation (1) and prevails at relatively high fields, such that:

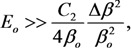
(61)
while the contribution of the second factor becomes notable at low fields obeying the opposite condition. Representation of Equation (61) in standard Fowler-Nordheim form results in:

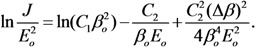
(62)

As is seen the right part of this equation contains along with the linear also squared dependence on the inversed electrical field strength 1/*Е_о_*.

**Figure 16:**
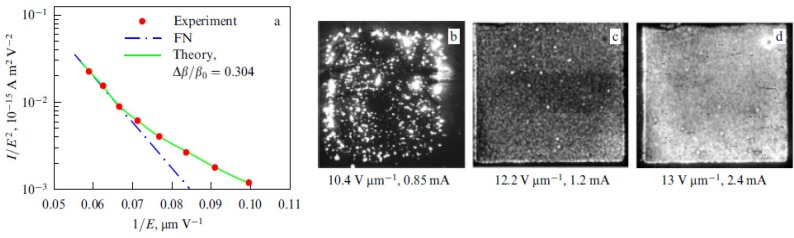
Illustration of the influence of the statistical spread of CNT parameters on the operating characteristics of a field emission cathode: (**a**) comparison of the Fowler-Nordheim characteristic (dashed-dotted line) with the calculated results obtained on the basis of generalized Equation (62) (solid line), and with the measured data [86] (dots); (**b**)–(**d**) the images of the distribution of the luminescence intensity over the phosphor surface, obtained at various values of the electric field strength and the emission current [86].

The above-derived dependence [Equation (62)] was used for treating the current-voltage characteristics of CNT-based field emission cathodes with the aim of determination of the degree of homogeneity of parameters of individual emitters. The typical current-voltage characteristic of such a kind is presented on Figure 16a in Fowler-Nordheim coordinates [86]. As is seen in a comparatively high field region this dependence has practically rectilinear form which is in agreement with the Fowler-Nordheim Equation (1). In a low field region this characteristic shows a notable deviation from Equation (1) which is caused by the statistical spread of parameters of CNTs. The measured data fit the Equation (62) at Δβ/β_о_ = 0.304. The effect of statistical spread in the nanotubes emission properties upon the current-voltage characteristics of a CNT-emitter can be seen directly from the comparison of distributions of the intensity of luminescence over the anode surface obtained at various magnitudes of the applied voltage and presented on Figure 16b,d. As is seen an increase in the electrical field strength results in not only enhancement of the luminescence brightness but also rise in the area of shining surface.

The above-performed analysis has indicated that due to the natural statistical non-homogeneity of a CNT array a relative quantity of nanotubes contributing into the emission current depends on the electrical field strength. At low fields only few of the most protruding nanotubes having quite high magnitude of the electrical field strength can emit electrons. As the field enhances the number of such nanotubes increases so the nanotube with a moderate value *β* are able to contribute into the emission. At very high fields practically all the nanotubes are involved into the emission. These considerations have been confirmed directly in the experiment [86] where single walled nanotubes synthesized in an electrical arc discharge using Ni/Cr catalyst [87] were used as a source of the emission.

The above-derived inter-relation between the shape of the current-voltage characteristic of a CNT-based cathode and a relative dispersion of parameters of individual emitters permits determination of the degree of non-homogeneity of a CNT array by processing the current-voltage characteristic. Table 3 presents the results of such a processing performed for several current-voltage characteristics of CNT-based field emission cathodes measured by various authors. As is seen the magnitude of the relative spread of the parameter *β* determined by processing various measurement data ranges within the region Δ*β*/*β* = 0.1 − 0.3. Taking into account a sharp exponential dependence of the emission current from this parameter one can conclude that the cathodes under consideration are characterized by rather high degree of the surface non-homogeneity. Note that the above-stated inter-relation between the degree of non-homogeneity of a cathode and the shape of its current-voltage characteristic can be used for analysis of the quality of a CNT array designed for a purpose not necessary related to the field emission.

**Table 3:** Relative dispersion of the electrical field amplification factor Δ*β*/*β* determined through treating the experimental data for CNT-based electron field emission cathodes [31].

The type of CNTs	Diameter (nm)	Emitters density (cm^−2^)	Inter-electrode gap (mm)	Δ *β*/*β*	Voltage range (kV)	Current range (μA)	Ref.
SW *	5	10^5^	5–20	0.24	5–15	10^−4^–10^2^	[88]
SW	1–2	10^5^	0.006	0.16	0.01–0.02	10^−4^–10^2^	[89]
Unknown		10^5^	0.002	0.105	0.02–0.07	0.1–5	[90]
SW	1.2		0.25	0.103	0.2–0.4	10^−6^–1	[91]
MW	25		0.25	0.13	0.4–0.7	10^−6^–0.1	[91]
MW	20		1	0.18	0.02–0.04	0.1–5	[92]
SW	1–1.5		0.5	0.304	0.1–9	10^−2^–10^2^	[31]

Notes: * SW is single walled nanotubes; MW is multi-walled nanotubes.

The statistical spread of the emission properties of individual CNTs can be caused not only by a difference in their geometric parameters, but also by differences in the angles of inclination of nanotubes with respect to the substrate surface. In this case, as was shown above [see Equation (9) and Figure 7], the electric field amplification factor of an individual CNT depends on the angle of inclination [30]. This can promote a deviation of the current-voltage characteristic of the cathode from the Fowler-Nordheim Equation (1), so that the degree of deviation is determined by both the spread of the angles of CNT inclination and the average value of this parameter.

The current-voltage characteristics of a CNT-based cathode, taking account of the statistical spread in the angles of inclination, were calculated in [30]. In so doing, the above Equation (9) of the electric field amplification factor on the angle of inclination of the nanotube were used. The tilt angle distribution of CNTs was supposed to obey the Poisson function:

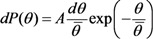
(63)
where *θ* is the average value of the inclination angle and the factor:

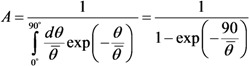
(64)
is determined by the normalization equation:

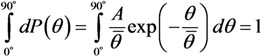
(65)

In the case of *θ* << 1 the value *A* is close to unity. Thus *A*(*θ* = 20°) = 1.011. The emission current density for a CNT array with randomly distributed inclination angle is expressed through the obvious integral:

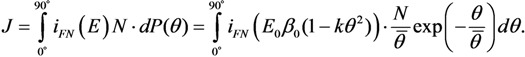
(66)

Here *i*_FN_(*E*) is the emission current of an individual nanotube determined by the Fowler-Nordheim Equation (1) with taking account the dependence of the electrical field amplification factor on the inclination angle [Equation (9)] and the random distribution of CNTs over the inclination angle Equation (28), *N* is the number of nanotubes per the unit area of the emitter.

**Figure 17:**
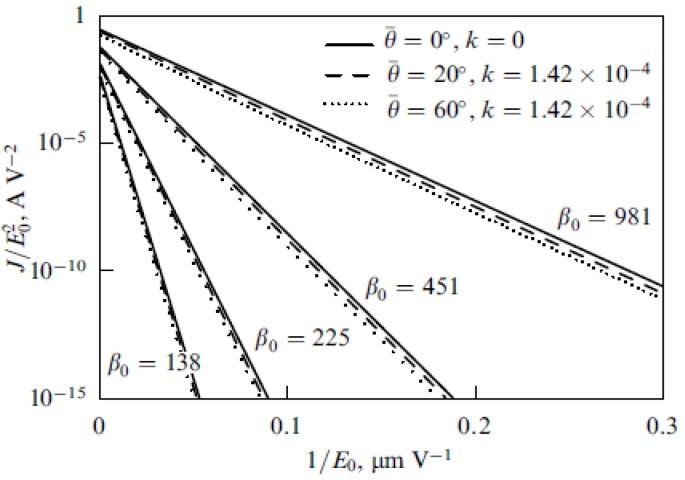
Current-voltage characteristics of a CNT-based cathode calculated taking into account the nanotube's inclination according to Equation (66) [30]. The case disregarding the inclination effect corresponds to *θ* = 0.

Figure 17 presents the current-voltage characteristics of a cathode calculated on the basis of Equation (66) with taking account the spread of CNTs over the inclination angle for various values *θ*. As is seen even for a large magnitude *θ* = 60° the deviation of the calculated dependence from the Fowler-Nordheim function is rather small comparing to that related to the statistical spread in geometry parameters of CNTs (compare Figure 16а).

### 3.3. Field-Induced Alignment and Current-Voltage Characteristics

The influence of the electric field on the degree of the CNT vertical alignment changes the current-voltage characteristics of the cathode. Indeed, an increase in the potential applied to the cathode not only causes an increase in the emission current in accordance with the Fowler-Nordheim expression, but also leads to an increase in the electric field amplification factor due to a change of the CNT angle of orientation relative to the substrate plane [30]. Thus, the shape of the *I*–*V* characteristics of the nanotube array whose initial orientation angle differs from nonzero can differ from the classic Fowler-Nordheim dependence. The magnitude of this difference is determined by the array geometry and mechanical characteristics of the nanotubes substituting it.

**Figure 18:**
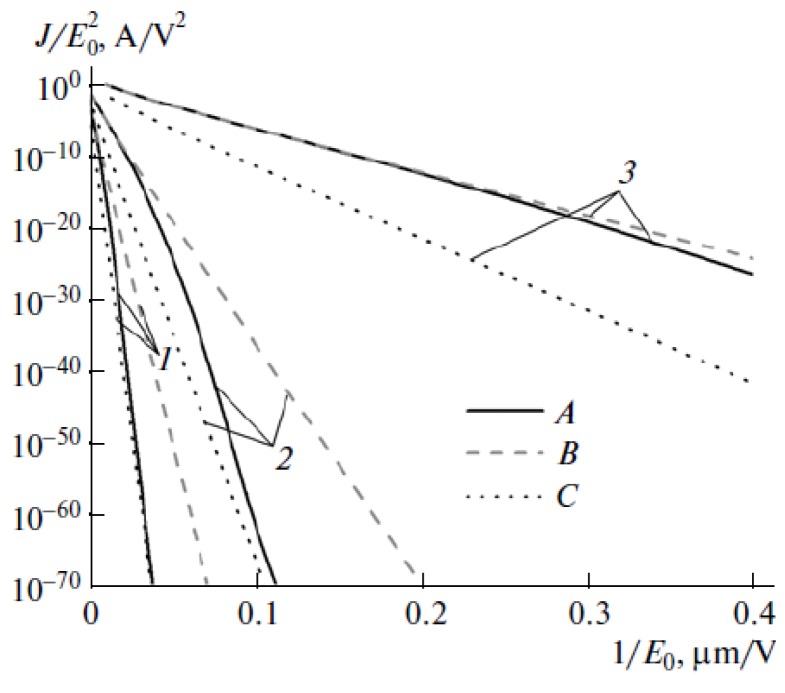
Current-voltage characteristics of the arrays containing 1000 CNTs of length *L*, nm: 100–300 (**1**), 500–800 (**2**), 1000–5000 (**3**), with diameter *D* = 10–50 nm and the Young modulus in the range *Y* = 10–50 GPa. The initial angle of inclination of the CNT was selected from the range *θ* = 50°–80°; *A* is the result of computations based on Equations (1), (9) and (44); *B* is the result of computation under the assumption that all nanotubes are aligned vertically; and *C* is the result of computation disregarding the effect of field orientation.

To illustrate the influence of the electric field on the emission properties of the cathode based on the CNTs, the *I*–*V* characteristics of an array containing 1000 CNTs inclined relative to the substrate plane were analyzed [68]. The angle of inclination and the geometrical parameters of the nanotubes are distributed at random in a certain range of values. In our computations, we used the angular dependence of the amplification factor [Equation (9)], while the dependence of angle of inclination *θ* on electric field strength *E* was determined from balance Equation (44) (Figure 8). Figure 18 shows the results of computations of the dependences of the nanotube angle of bending under the action of the electric field on the average value of field strength *E*. The computations were performed for CNTs of different lengths, divided into three groups. The CNT parameters were chosen at random with the help of a random number generator from a certain range of values which are given in Table 4. The results of computations are compared in Figure 18 with the *I*–*V* characteristics computed using the Fowler-Nordheim expression disregarding the field influence on the nanotube alignment, as well as with the corresponding dependences obtained for an array of vertically aligned nanotubes. The computations were carried out disregarding the screening effect and assuming that the individual CNTs are separated by appreciable distances from one another. The value of the electron work function was supposed to be equal to *φ_o_* = 4.6 eV.

It can be seen from the computations that the influence of the electric field on the CNT emission properties is most significant for relatively low fields, in which orienting action of the field involves a maximal number of the nanotubes. The fraction of vertically aligned CNTs increases with the electric field strength, so that the *I*–*V* characteristic approaches the dependence typical of the array of vertically aligned CNTs. The longer the nanotube, the closer its *I*–*V* characteristic to the dependence typical of vertically aligned emitters. The computed dependences reach a saturation which magnitude is determined by the geometrical parameters of the nanotube, as well as by its mechanical properties (the Young modulus).

**Table 4:** Parameters of the nanotubes for which the computations represented in Figure 18 are performed.

No.	*D* (nm)	*θ* (°)	*Y* (GPa)	*L* (μm) (0.1–0.3)	*L* (μm) (0.5–0.8)	*L* (μm) (1–5)
1	49.3	66.3	36.9	0.183	0.754	1.88
2	45.8	59.1	45.2	0.286	0.695	2.76
3	46.8	60.2	18.9	0.252	0.730	2.80
4	27.9	53.6	34.7	0.214	0.573	2.57
5	38.7	71.1	19.8	0.214	0.524	2.07
6	19.5	61.4	44.2	0.109	0.602	3.66
7	22.2	57.1	47.4	0.237	0.674	1.62
8	27.1	66.3	30.5	0.216	0.633	1.98
9	10.4	57.4	49.7	0.153	0.732	1.87
10	15.5	63.6	43.0	0.206	0.524	3.70
11	42.7	57.4	25.6	0.21	0.702	1.79

### 3.4. Self Electric Field of Nanotubes

The nanotubes constituting the emission cathode are located on a conducting substrate. The work function of the substrate material naturally differs from the CNT work function; therefore, a contact potential difference (CPD), which determines a nonzero potential of the nanotube relative to the substrate, is formed between the substrate and the nanotube. The value of this potential is relatively low (at a level of tenths of a volt); however, due to the miniature size of the nanotube, this potential gives rise to sufficiently high electric fields up to ~10^4^ V/cm. The above-given estimates demonstrate that such fields can affect the angle of orientation of the nanotubes. Even in the case of the array consisting of the initially vertically aligned CNTs, the self electric field of the nanotubes produced by CPD can change their alignment, which affects the shape of current–voltage emission characteristics of the cathode.

**Figure 19:**
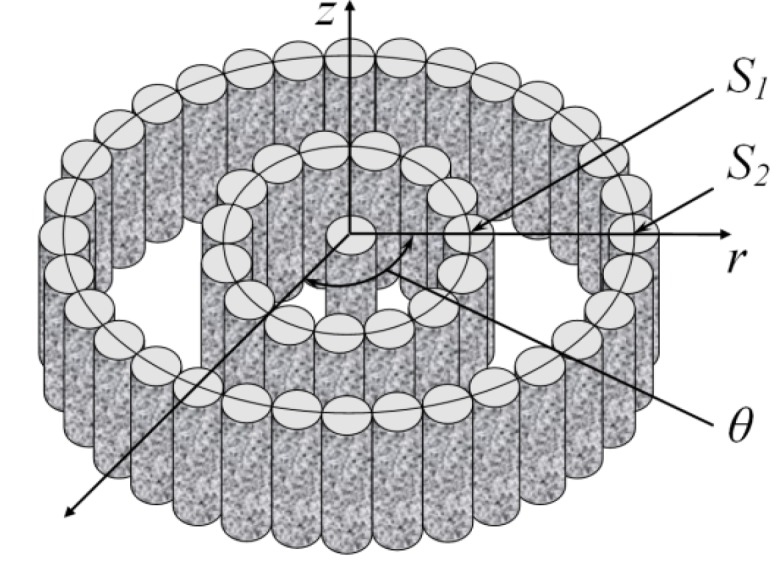
Model configuration of the CNT array under consideration. Here (*r*, *θ*, *z*) are the cylindrical coordinates; *S*_1_ and *S*_2_ are the distances from the CNT rows to the array center.

In order to find the influence of the self electric field of the nanotubes, related to the CPD, on their alignment in the array, we considered a model configuration of the CNT array shown in Figure 19 [68]. In this configuration, the rows of similar vertically aligned CNTs are located on a substrate on concentric circles separated by distance *S* = *S*_2_ – *S*_1_. Due to the CPD, the tubes are repelled from one another irrespective of the CPD sign, which leads to the dependence of the angle of orientation on the distance to the center. The larger the distance to the center, the greater the value of the repulsive force acting on the CNT in the direction from the center to the periphery.

**Figure 20:**
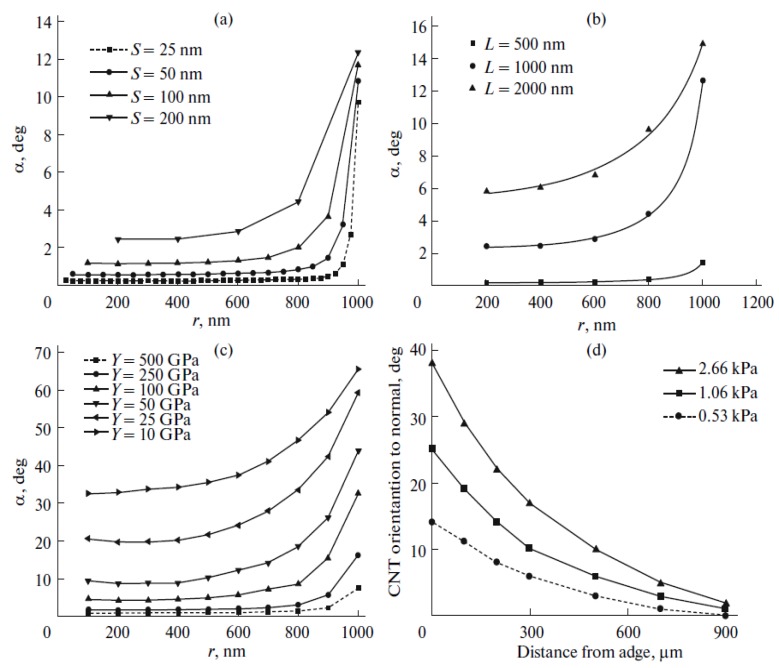
Dependences of the CNT orientation angle on the position on the substrate: (**a**) on the distance to the center, computed for the arrays with different distances *S* between the rows under the assumption that the contact potential difference between the CNT and the substrate material is Δ*φ* = 1 eV; the CNT diameter *D* = 10 nm, length *L* = 1 μm, Young modulus *Y* = 30 GPa; (**b**) the same dependences computed for the CNT arrays with Young modulus *Y* = 30 GPa, diameter *D* = 10 nm, and various lengths, spacing *S* between the rows being 200 nm; (**c**) the same dependences, computed for the CNT arrays with different Young moduli; and (**d**) the dependences of the CNT orientation angle on the distance to the array edge, which were measured for the nanotubes synthesized under different pressures of the buffer gas [93].

Figure 20a–c show the dependences of the CNT angle of orientation on the distance to the array center, which were computed using the solutions to mechanical balance Equation (44) and to the Laplace equation for the CNT array. These computations were made for the CNT arrays of different geometries and with different mechanical characteristics. Each of the arrays has a structure shown in Figure 19 and contained ten rows of CNTs equidistant from each other. For the sake of comparison, the dependence of the CNT angle of orientation on the distance to the array periphery, measured in [93], is shown in Figure 20d. As can be seen from the figure, the predicted effect of the action of CNTs constituting the array on one another, which leads to the dependence of the angle of orientation on the position relative to the array center, has found a qualitative experimental confirmation. The quantitative comparison of the computation results with the experiments requires the information on the mechanical characteristics (Young modulus) of the CNTs constituting the array. In addition, there are no data on the geometry of nanotubes in [93]. It should be noted that a descending dependence of the angle of orientation on the distance to the array edge was also observed in [70], in which the authors used the array of vertically aligned carbon filaments as the emission source.

As can be seen from the results of computations, the CNT angle of inclination increases with the nanotube height (*i.e.*, the higher the nanotube, the more bendable it is). The dependence of the angle of inclination on the spacing between the rows in the array can be considered as a manifestation of the effect of screening the electric field by the neighboring nanotubes [30,47,49]. The closer the nanotubes packing, the lower the electric field amplification factor, which determines the value of the local electric field near the CNT tip. As the distance from the array center increases, the screening effect becomes weaker, which increases the angle of inclination of CNTs.

**Figure 21:**
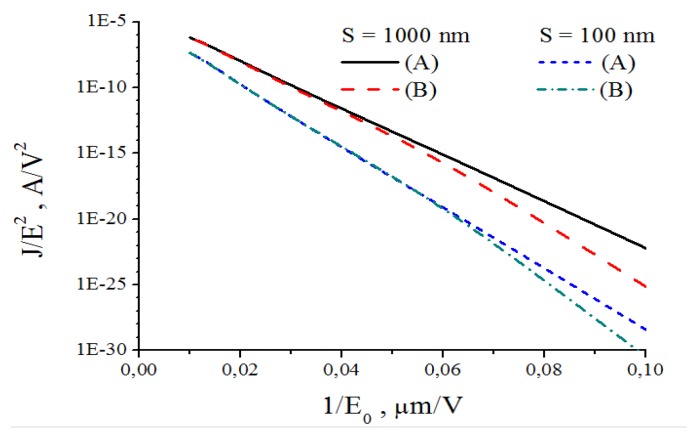
Current–voltage characteristics of the CNT cylindrical array (see Figure 19), computed disregarding (A) and taking into account (B) the self electric field of the nanotubes for the distances *S* = 1000 and 100 nm between the rows. The height of the nanotubes *L* = 1 μm, diameter *D* = 5 nm, electron work function *φ* = 4.6 eV, and Young modulus *Y* = 50 GPa. Contact potential difference Δ*φ* = 1 V. The array radius in both cases is equal to 10 μm, which corresponds to the number of rows 10 and 100, respectively, in the array.

If the external electric field is applied to induce the electron emission, the role of the self electric field associated with CPD becomes smaller. The CNT angle of inclination decreases with an increase in the external field, which affects the shape of the current–voltage characteristics of the array. The influence of the self electric field on the emissivity of the CNT array is illustrated in Figure 21, where the current–voltage characteristics of the CNT array having the structure shown in Figure 19 computed with and without regard to this influence are compared. It can be seen that allowance for the self electric field of the nanotubes, which is caused by the CPD, reduces the emission current; this effect is most noticeable in the range of low values of the electric field. This effect almost disappears in case of strong fields with a value significantly exceeding the self electric field of a CNT.

### 3.5. Optimization of Parameters of a CNT–Based Field Emission Cathode

As it was noted above (see Section 3.1. and Figure 15), the dependence of the emission current density of a CNT array on the inter-tube spacing *S* has a non-monotone shape. Such a peculiarity is caused by the screening effect, which promotes a decrease in the electrical field enhancement factor as the inter-tube spacing decreases. This effect forms the basis for the optimization of a CNT electron field emitter in terms of the maximum electron current density [28]. However, the simplest version of the optimization procedure developed in [28] requires for further modification. Firstly, due to the non-linear character of the Fowler-Nordheim Equation (1), the optimum inter-tube distance providing the maximum electron current density depends on the applied voltage. Secondly, the optimization procedure should take into account the phenomenon of thermal instability limiting the emission current of an individual nanotube (see Section 2.3.3.). The instability threshold depends on the nanotube’s height *h*, therefore the optimum geometry of a CNT array is shifted with taking into consideration this phenomenon. The instability mechanism is caused by a sharply increasing dependence of the emission properties of a nanotube from the tip temperature. Heat is released through heat conduction, whose intensity is proportional to the tip temperature, and the heat release rate is characterized by significantly sharper temperature dependence. Therefore, the heat balance in the nanotube is violated at a certain threshold emission current. As a result, the tip temperature increases in an unlimited manner and the nanotube undergoes thermal failure. The thermal instability conditions are determined by solving a heat conduction equation for a nanotube, and its stationary solution exists only in a certain emission current range [57]. Since instability conditions are more easily met for long and thin nanotubes (heat release from them is hindered), the thermal instability changes the optimum parameters of the cathode. The optimization procedure involving both screening effect with taking into account the nonlinear Fowler-Nordheim Equation (1) and the thermal instability of a CNT-based emitter has been developed in [94].

The procedure of optimizing the emission properties of a CNT array includes the solution of an electrostatic problem, which can determine the screening effect, and the solution of a heat conduction equation for a nanotube, which can determine the maximum emission current limited by the development of thermal instability. Moreover, we use the Fowler-Nordheim relationship, which relates the emission current to the voltage at a nanotube tip. The optimization procedure consists of the following steps.

(1) Emission current *I* is specified for a nanotube of certain geometry.
(2) A heat conduction Equation (10) is solved for a nanotube of length *h* at specified emission current *I*, following the approach described in Section 2.3.
(3) The relationship between critical emission current *I_c_* and local electric field strength *E* at a CNT tip is described by the Fowler-Nordheim Equation (1) containing the tip temperature as a parameter.
(4) At a fixed average electric field strength *E*_o_ and local electric field *E* determined by Equation (66), we find the electric field enhancement factor *β* with taking into consideration the screening effect,
  *E* = *в*(*S*, *h*, *d*)*E*_o_ (67)
  where *d* is the nanotube’s diameter.
(5) We take into account the dependence of the electrical field enhancement factor on the nanotube geometry and inter-tube spacing *S* found earlier from the solution to a Laplace equation (Figure 15a) and determine optimum distance *S*_o_ corresponding to the given nanotube geometry (*d*, *h*). Obviously, the maximum emission current density is:

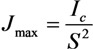
(68)

**Figure 22:**
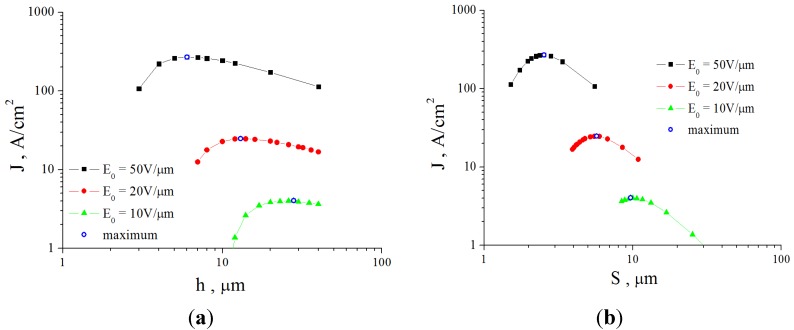
Dependences of the emission current density of a CNT array on (**a**) the nanotube height, and (**b**) the inter-tube distance calculated for various average electric field strengths. *E*_o_ = 50 (■), 20 (●), and 10 (▲) V/μm; (○) maximum value [94].

Figure 22 shows the dependences of the emission current density of a CNT array on the nanotube height and the inter-tube distance calculated for various average electric field strengths. These dependences were obtained using the procedure described above with taking into account the screening effect and the thermal instability of nanotubes. The nanotube diameter was taken to be *d* = 10 nm, the work function was *φ* = 4.6 eV, and the parameters characterizing the temperature dependences of the transport coefficients were *l_fp_* = *l_fe_* = 480 nm and *α* = 1.5.

**Figure 23:**
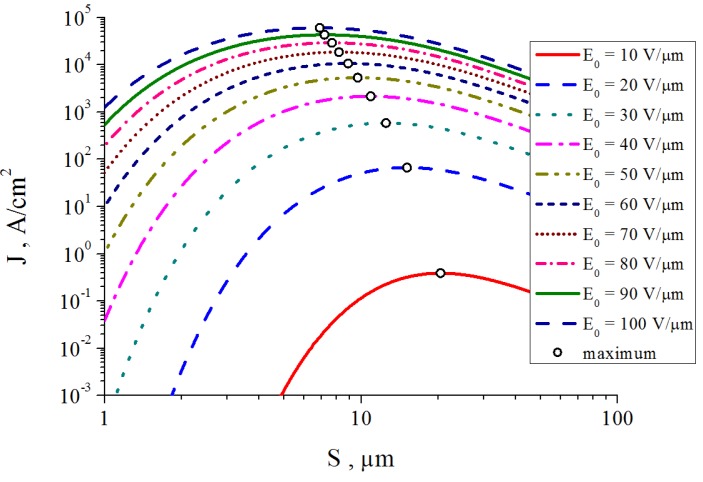
Dependences of the emission current density on the inter-tube distance calculated at various average electric field strengths with taking into account the screening effect and thermal instability: (□) points with a maximum in the *J*(*S*) dependences, (▼) points where thermal instability appears, and (Δ) optimized *J*(*S*) curve for a certain CNT array. The nanotube diameter is *d* = 10 nm and the nanotube height is *h* = 10 μm. *E*_o_ = (**1**) 11.4, (**2**) 20, (**3**) 30, (**4**) 40, (**5**) 50, (**6**) 60, (**7**) 70, (**8**) 80, (**9**) 90, and (**10**) 100 V/μm.

The calculation results shown in Figure 22 indicate a monotonic descending dependence of the optimum inter-tube distance on the average electric field strength. A decrease in the optimum inter-tube distance is naturally accompanied by an increase in the maximum emission current density with the applied voltage. More detailed calculations of this type are represented in Figure 23, where a wider average electric field range is covered.

**Figure 24:**
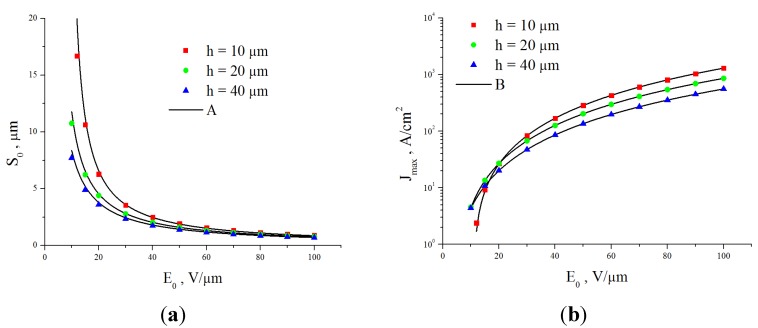
Dependences of (**a**) optimum internanotube distance *S*_o_ and (**b**) maximum emission current density *J*_max_ on average electric field *E*_o_ calculated for CNTs of diameter = 10 nm and various heights. Solid lines: Approximation by Equations (69) and (70) at *h* = 10 (■), 20 (●), and 40 (▲) μm.

Figure 24 presents the dependences of the optimum inter-tube distance and the maximum emission current density on the average electric field calculated for nanotubes of various heights. These dependences can be approximated by the expressions:

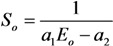
(69)

*J*_max_ = *E*_o_(*a*_3_*E*_o_ − *a*_4_) (70)

The values of parameters *a*_1_, *a*_2_, *a*_3_, and *a*_4_ are given in Table 5.

**Table 5:** The values of the fitting parameters involved into Equations (69) and (70) evaluated for *d* = 10 nm.

*h* (μm)	*a*_1_ (V^−1^)	*a*_2_ (μm^−1^)	*a*_3_ (Aּμm^2^/cm^2^ּV^2^)	*a*_4_ (Aּμm/Vּcm^2^)
10	0.012	0.085	0.146	1.608
20	0.013	0.040	0.090	0.463
40	0.015	0.019	0.057	0.135

**Figure 25:**
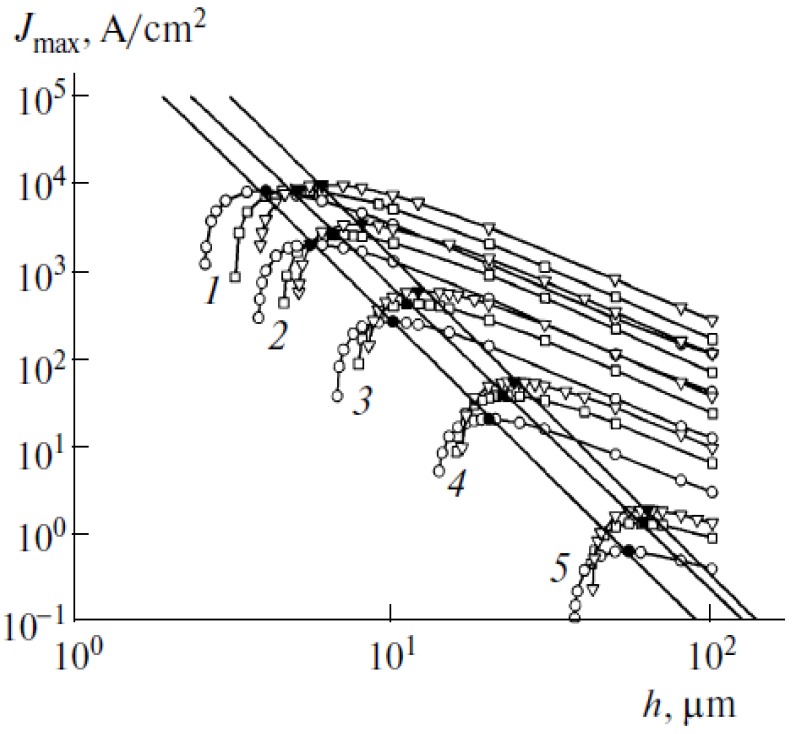
Optimization curves calculated for an array of CNTs of height *h* and various diameters *d* calculated under different assumptions regarding the absolute values and temperature dependences of the transfer coefficients: *d* = 1 (**1**), 2 (**2**), 5 (**3**), 14 (**4**), and 50 (**5**) nm. Approximation for (●) *α* = 1, *l_p_*= 100 nm; (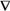) *α* = 1, *l_p_* = 480 nm; (■) *α* = 1.5, *l_f_* = 480 nm.

The above-presented approach [94] to optimizing the emission properties of CNT-based field emission cathodes has an uncertainty related to an arbitrary choice of the temperature dependences of the transfer coefficients of nanotubes. This uncertainty characterizes the scatter of the existing experimental and calculated data [59] and is mainly caused by a high sensitivity of the defect content in CNTs to synthesis conditions. To estimate the sensitivity of the optimization results to the choice of the transfer coefficients, we performed optimization calculations using various values of parameters *l_f_* (*l_f_* = *l_fp_* = *l_fe_*) and *α*, which characterize the absolute values and temperature dependences of the transfer coefficients in CNTs. A comparison of the results of these calculations performed for nanotubes of various sizes (Figure 25) indicates a relatively low sensitivity of the optimization results to the choice of the transfer coefficients in CNTs.

## 4. Conclusions

Development of the new direction of vacuum electronics involving CNT-based field emission cathodes requires for thorough theoretical analysis. Such an analysis performed by various groups during last decade has resulted in formulation of the main physical effects influencing the operation of both individual CNT-based sources and cathodes as a whole. The list of the effects includes basically the field enhancement phenomenon near a CNT tip; alignment and misalignment of CNTs experienced by the action of the electrical field; effect of field screening by neighboring nanotubes; thermal effects leading to degradation of nanotubes. These phenomena are inter-related with each other and the optimization procedure for a CNT-based cathode should be performed as a result of combined consideration of those. Such a combined consideration should be based on the solution of the set of equation including the Laplace equation for the distribution of the electrical potential in a vicinity of the CNT tip with taking into account the effect of screening the electrical field by neighboring nanotubes non-linear heat conduction equation for an emitting nanotube with taking into account the Ohmic heating of nanotube, thermal heat conduction removal and the temperature dependence of the emission ability of a nanotube; the Fowler-Nordheim equation inter-connecting the emission current and the applied voltage.

The contemporary state of calculation technique permits the numerical solution of the above-mentioned set of equations with considerable accuracy, however it is hardly possible to expect for matching between the theoretical calculation results and measurement data. The main reasons of such a mismatching are in a rather wide spread of electronic characteristics of nanotubes produced within the frame of the common growth procedure and in high sensitivity of those to the defect content and deviation from the vertical alignment. The structural defects affect the heat conduction and electric conduction coefficients, while possible tilting of nanotubes promotes lowering in the field enhancement factor, which magnitude becomes to be dependent on the applied voltage. Besides of that, the proper operation of a CNT-based filed emitter depends on the quality of the electrical contact between the conductive substrate and the nanotube. The performed measurements indicate that the contact resistance can exceed considerably the resistance of nanotube, so that the total resistance of the emitter is determined by the contact resistance which in its turn is very sensitive to the fabrication procedure and can not be fixed. Therefore, a CNT-based emitter is not so precisely determined object to be described theoretically with the accuracy sufficient to experimental matching. In such a situation the main value of the theory as applied to the development of CNT-based field emission cathodes is rather in establishing the main physical effects determining peculiarities of the cathode operation and in qualitative prediction of the main trends in behavior of the cathode in various conditions. The present article demonstrates some examples of such an approach indicating the role of various physical effects in operation of CNT-based cathodes.

## References

[1] Chernozatonskii L.A., Gulyaev Y.V., Kosakovskaja Z.J., Sinitsyn N.I., Torgashov G.V., Zakharchenko Yu, Fedorov E.A., Val’chuk V.P. (1995). Electron field emission from nanofilament carbon films. Chem. Phys. Lett..

[2] De Heer W.A., Chatelain A., Ugarte D. (1995). A carbon nanotube field-emission electron source. Science.

[3] Rinzler A.G., Hafner J.H., Nikolaev P., Nordlander P., Colbert D.T., Smalley R.E., Lou L., Kim S.G. (1995). Unraveling nanotubes: Field emission from an atomic wire. Science.

[4] Eletskii A.V. (2002). Carbon nanotubes and their emission properties. Phys. Usp..

[5] Eletskii A.V. (2010). Carbon nanotube-based electron field emitters. Phys. Usp..

[6] Sohn J.I., Lee S., Song Y.H., Choi S.-Y., Cho K.-I., Namet K.-S. (2001). Patterned selective growth of carbon nanotubes and large field emission from vertically well-aligned carbon nanotube field emitter arrays. Appl. Phys. Lett..

[7] Wang Q.H., Yan M., Chang R.P.H. (2001). Flat panel display prototype using gated carbon nanotube field emitters. Appl. Phys. Lett..

[8] Mauger M., Vu T.V. (2006). Vertically aligned carbon nanotube arrays for giant field emission displays. J. Vac. Sci. Technol. B.

[9] Matsumoto T., Mimura H. (2003). Point X-ray source using graphite nanofibers and its application to X-ray radiography. Appl. Phys. Lett..

[10] Yue G.Z., Qiu Q., Gao B., Cheng Y., Zhang J., Shimoda H., Chang S., Lu J.P., Zhou O. (2002). Generation of continuous and pulsed diagnostic imaging X-ray radiation using a carbon-nanotube-based field-emission cathode. Appl. Phys. Lett..

[11] Gutman G., Strumban E., Sozontov E., Jenrow K. (2007). X-ray scalpel—A new device for targeted X-ray brachytherapy and stereotactic radiosurgery. Phys. Med. Biol..

[12] Dickler A. (2009). Xoft Axxent electronic brachytherapy—A new device for delivering brachytherapy to the breast. Nat. Rev. Clin. Oncol..

[13] Schneider F., Fuchs H., Steil F.L.V., Ziglio F., Kraus-Tiefenbacher U., Lohr F., Wenzet F. (2009). A novel device for intravaginal electronic brachytherapy. Int. J. Radiat. Oncol. Biol. Phys..

[14] Rivard M.J., Davis S.D., De Werd L.A., Rusch Thomas W., Axelrod S. (2006). Calculated and measured brachytherapy dosimetry parameters in water for the Xoft Axxent X-Ray Source: An electronic brachytherapy source. Med. Phys..

[15] Kim H.J., Ha J.M., Heo S.H., Choy S.O. (2012). Small-sized flat-tip CNT emitters for miniaturized X-ray tubes. J. Nanomater..

[16] Zhang J., Yang G., Lee Y.Z., Lu J.P., Zhou O. (2006). Multiplexing radiography using a carbon nanotube based X-ray source. Appl. Phys. Lett..

[17] Kawakita K., Hata K., Sato H., Saito Y. (2006). Development of microfocused X-ray source by using carbon nanotube field emitter. J. Vac. Sci. Technol. B.

[18] Saito Y., Uemura S., Hamaguchi K. (1998). Cathode ray tube lighting elements with carbon nanotube field emitters. Jpn. J. Appl. Phys..

[19] Saito Y., Uemura S. (2000). Field emission from carbon nanotubes and its application to electron sources. Carbon.

[20] Obraztsov A.N., Kleshch V.I. (2009). Cold and laser stimulated electron emission from nanocarbons. J. Nanoelectron. Optoelectron..

[21] Croci M., Arfaoui I., Stöckli T., Chatelain A., Bonard J.-M. (2004). A fully sealed luminescent tube based on carbon nanotube field emission. Microelectron. J..

[22] Antony J., Qiang Y. (2007). Cathodoluminescence from a device of carbon nanotube-field emission display with ZnO nanocluster phosphor. Nanotechnology.

[23] Bonard J., Stöckli T., Noury O., Châtelain A. (2001). Field emission from cylindrical carbon nanotube cathodes: Possibilities for luminescent tubes. Appl. Phys. Lett..

[24] Teo K.V.K., Minoux E., Hudanski L., Peauger F., Schnell J.-P., Gangloff L., Legagneux P., Dieumegard D., Amaratunga G.A.J., Milneet W.I. (2005). Microwave devices: Carbon nanotubes as cold cathodes. Nature.

[25] Milne W.I., Teo K.B.K., Minoux E., Groening O., Gangloff L., Hudanski L., Schnell J.-P., Dieumegard D., Peauger F., Bu I.Y.Y. (2006). Aligned carbon nanotubes/fibers for applications in vacuum microwave amplifiers. J. Vac. Sci. Technol. B.

[26] Fowler R.H., Nordheim L. (1928). Electron emission in intense electric fields. Proc. R. Soc. Lond. A.

[27] Gomer R. (1993). Field Emission and Field Ionization.

[28] Nilsson L., Groening O., Emmenegger C., Kuettel O., Schaller E., Schlapbach L., Kind H., Bonard J.-M., Kern K. (2000). Scanning field emission from patterned carbon nanotube films. Appl. Phys. Lett..

[29] Bocharov G.S., Eletskii A.V. (2005). Effect of screening on the emissivity of field electron emitters based on carbon nanotubes. Tech. Phys..

[30] Belsky M., Bocharov G., Eletskii A., Sommerer T. (2010). Field amplification in carbon nanotube’s based field emission cathodes. Tech. Phys..

[31] Bocharov G.S., Eletskii A.V., Korshakov A.V. (2003). Emission characteristics of carbon nanotube-based cathodes. Rev. Adv. Mater. Sci..

[32] Zou R., Hu J., Song Y., Chen H., Chen H., Wu J., Sun Y., Chen Z. (2010). Carbon nanotubes as field emitter. J. Nanosci. Nanotechnol..

[33] Saito R., Dresselhaus G., Dresselhaus M. (1998). Physical Properties of Carbon Nanotubes.

[34] Dresselhaus M.S., Dresselhaus G., Avouris P. (2001). Carbon Nanotubes: Synthesis, Structure, Properties and Applications.

[35] Dobretsov L.N., Gomoyunova M.V. (1966). Emission Electronics.

[36] Luo J., Peng L.M., Xue Z.Q., Wu J.L. (2002). End potential barriers of single-walled carbon nanotubes and their role in field emission. Phys. Rev. B.

[37] Han S., Ihm J. (2002). First-principles study of field emission of carbon nanotubes. Phys. Rev. B.

[38] Qiao L., Wang C., Qu C., Zeng Y., Yu S.S., Hu X.Y., Zheng W.T., Jiang Q. (2009). First-principles investigation on the field emission properties of B-doped carbon nanotubes. Diamond Relat. Mater..

[39] Zheng X., Chen G., Li Z., Xu N. (2004). Quantum-mechanical investigation of field-emission mechanism of a micrometer-long single-walled carbon nanotube. Phys. Rev. Lett..

[40] Yaghoobi P., Walus K., Nojeh A. (2009). First-principles study of quantum tunneling from nanostructures: Current in a single-walled carbon nanotube electron source. Phys. Rev. B.

[41] Bulashevich K.A., Rotkin V.V. (2002). Nanotube-based devices: Microscopic model. JETP Lett..

[42] Mishchenko E.G., Raikh M. (2006). Electrostatics of straight and bent single-walled carbon nanotubes. Phys. Rev. B.

[43] Li Z.B., Wang W.L. (2006). Analytic solution of charge density of single wall carbon nanotube under conditions of field electron emission. Chin. Phys. Lett..

[44] Sedrakyan T.A., Mishchenko E.G., Raikh M.E. (2006). Penetration of external field into regular and random arrays of nanotubes: Implications for field emission. Phys. Rev. B.

[45] Zhao G., Zhang J., Zhang Q., Zhang H., Zhou O., Qin L.-C., Tang J. (2006). Fabrication and characterization of single carbon nanotube emitters as point electron sources. Appl. Phys. Lett..

[46] Collins P.G., Zettle A. (1997). Unique characteristics of cold cathode carbon-nanotube-matrix field emitters. Phys. Rev. B.

[47] Cheng Y., Zhou O. (2003). Electron field emission from carbon nanotubes. C. R. Phys..

[48] Kokkorakis G., Modinos A., Xanthakis J.P. (2002). Local electric field at the emitting surface of a carbon nanotube. J. Appl. Phys..

[49] Eletskii A.V., Bocharov G.S. (2009). Emission properties of carbon nanotubes and cathodes on their basis. Plasma Sources Sci. Technol..

[50] González-Berríos A., Piazza F., Morell G. (2005). Numerical study of the electrostatic field gradients present in various planar emitter field emission configurations relevant to experimental research. J. Vac. Sci. Technol. B.

[51] Xu Z., Bai X.D., Wang E.G., Wang Z.L. (2005). Field emission of individual carbon nanotube with in situ tip image and real work function. Appl. Phys. Lett..

[52] Edgcombe C.J., Valdrè U. (2001). Microscopy and computational modelling to elucidate the enhancement factor for field electron emitters. J. Microsc..

[53] Edgcombe C.J., Valdrè U. (2002). Experimental and computational study of field emission characteristics from amorphous carbon single nanotips grown by carbon contamination. I. Experiments and computation. Philos. Mag. B.

[54] Xu Z., Bai X.D., Wang E.G. (2006). Geometrical enhancement of field emission of individual nanotubes studied by *in situ* transmission electron microscopy. Appl. Phys. Lett..

[55] Martinson T., Malov Y.I. (2002). Differential Equations of Mathematical Physics.

[56] Vlasova E., Zarubin V., Kuvyrkin G. (2001). Approximate Methods of Mathematical Physics.

[57] Bocharov G.S., Eletskii A.V. (2007). Thermal instability of field emission from carbon nanotubes. Tech. Phys..

[58] Vincent P., Purcell S.T., Journet C., Binh V.T. (2002). Modelization of resistive heating of carbon nanotubes during field emission. Phys. Rev. B.

[59] Eletskii A.V. (2009). Transport properties of carbon nanotubes. Phys. Usp..

[60] Kim P., Shi L., Majumdar A., McEuen P.L. (2001). Thermal transport measurements of individual multiwalled nanotubes. Phys. Rev. Lett..

[61] Yi W., Lu L., Zhang D.-L., Pan Z.W., Xie S.S. (1999). Linear specific heat of carbon nanotubes. Phys. Rev. B..

[62] Gao B., Komnik A., Egger R., Glattli D.C., Bachtold A. Evidence for Luttinher-Liquid Behavior in Crossed Metallic Single-Wall Nanotubes. Proceeding of the NT'05: 6th International Conference on the Science and Application of Nanotubes.

[63] Sveningsson M., Hansen K., Svensson K., Olsson E., Campbell E.E.B. (2005). Quantifying temperature-enhanced electron field emission from individual carbon nanotubes. Phys. Rev. B.

[64] Huang N., She J.C., Chen J., Deng S.Z., Xu N.S., Bishop H., Huq S.E., Wang L., Zhong D.Y., Wang E.G. (2004). Mechanism responsible for initiating carbon nanotube vacuum breakdown. Phys. Rev. Lett..

[65] Bonard J.M., Maier F., Stöckli T., Châtelain A., de Heer W.A., Salvetat J.-P., Forró L. (1998). Field emission properties of multiwalled carbon nanotubes. Ultramicroscopy.

[66] Bonard J.M., Klinke C., Dean K.A., Coll B.F. (2003). Degradation and failure of carbon nanotube field emitters. Phys. Rev. B.

[67] Tang H., Liang S.D., Deng S.Z., Xu N.S. (2006). Comparison of field and thermionic emissions from carbon nanotubes. J. Phys. D.

[68] Bocharov G.S., Knizhnik A.A., Eletskii A.V., Sommerer T.J. (2012). Influence of the electric field on the alignment of carbon nanotubes during their growth and emission. Tech. Phys..

[69] Eletskii A.V., Kuzmany H., Fink J., Mehring M., Roth S. (2000). Growth of Elongated Structures in a Longitudinal Electrical Field. Electronic Properties of Novel Materials—Molecular Nanostructures.

[70] Merkulov V.I., Melechko A.V., Guillorn M.A., Simpson M.L., Lowndes D.H., Whealton J.H., Raridon R.J. (2002). Controlled alignment of carbon nanofibers in a large-scale synthesis process. Appl. Phys. Lett..

[71] Merkulov V.I., Melechko A.V., Guillorn M.A., Lowndes D.H., Simpson M.L. (2001). Alignment mechanism of carbon nanofibers produced by plasma-enhanced chemical-vapor deposition. Appl. Phys. Lett..

[72] Chhowalla M., Teo K.B.K., Ducati C., Rupesinghe N.L., Amaratunga G.A.J., Ferrari A.C., Roy D., Robertson J., Milne W.I. (2001). Growth process conditions of vertically aligned carbon nanotubes using plasma enhanced chemical vapor deposition. J. Appl. Phys..

[73] Shao-Jie M.A., Guo W.L. (2008). Mechanism of carbon nanotubes aligning along applied electric field. Chin. Phys. Lett..

[74] Bocharov G.S., Eletskii A.V. (2012). Degradation of a CNT-based field emission cathode due to ion sputtering. Fuller. Nanotub. Carbon Nanostruct..

[75] Bocharov G.S., Eletskii A.V. (2012). Degradation of a carbon nanotube-based field-emission cathode during ion sputtering. Tech. Phys..

[76] Itikawa Y.I. (2006). Cross sections for electron collisions with nitrogen molecules. J. Phys. Chem. Ref. Data.

[77] Itikawa Y.I. (2009). Cross sections for electron collisions with oxygen molecules. J. Phys. Chem. Ref. Data.

[78] Eckstein W. (2001). Atomic and Plasma-Material Interaction Data for Fusion.

[79] Kim W.J., Lee J.S., Lee S.M., Song K.Y., Chu C.N., Kim H. (2011). Better than 10 mA Field emission from an isolated structure emitter of a metal oxide/CNT composite. ACS Nano.

[80] Guglielmotti V.V., Tamburri E., Orlanducci S., Terranova M.L., Rossi M., Notarianni M., Fairchild S.B., Maruyama B., Behabtu N., Young C.C. (2013). Macroscopic self-standing SWCNT fibres as efficient electron emitters with very high emission current for robust cold cathodes. Carbon.

[81] Navitski A., Serbun P., Müller G., Joshi R.K., Engstler J., Schneider J.J. (2012). Role of height and contact interface of CNT microstructures on Si for high current field emission cathodes. Eur. Phys. J. Appl. Phys..

[82] Su W.S., Chuang F.C., Cho T.H., Leung T.C. (2009). The screening effect on field enhancement factor of the finite-length small radius single-walled carbon nanotubes. J. Appl. Phys..

[83] Smith R.C., Silva S.R.P. (2009). Interpretation of the field enhancement factor for electron emission from carbon nanotubes. J. Appl. Phys..

[84] Bocharov G.S., Eletskii A.V., Sommerer T.J. Optimization of the Parameters of a Carbon Nanotube-Based Field Emission Cathode. Proceedings of the 11th International Conference on the Science and Application of Nanotubes.

[85] Obraztsov A., Volkov A., Zakhidov A., Petrushenko Y.V., Satanovskaya O.P. (2003). Fundamental aspects and applications of low field electron emission from nanocarbons. Surf. Eng..

[86] Bocharov G.S., Eletskii A.V., Pal A.F., Pernbaum A.G., Pichugin V.V., Kuzmany H., Fink J., Mehring M., Roth S. (2004). Emission Characteristics of CNT-Based Cathodes. Electronic Properties of Synthetic Nanostructures.

[87] Bezmelnitsyn V.N., Domantovskii A.G., Eletskii A.V., Obraszova E.D., Pal A.F., Pernbaum A.G., Pichugin V.V., Prichod’ko K.E., Suetin N.V., Terekhov S.V. (2002). Production of single walled nanotubes with Ni/Cr as catalyst. Phys. Solid State.

[88] Yoshimoto T., Iwata T., Minesawa R., Matsumoto K. (2001). Emission properties from carbon nanotube field emitter arrays (FEAs) grown on si emitters. Jpn. J. Appl. Phys..

[89] Matsumoto K., Kinosita S., Gotoh Y., Uchiyama T., Manalis S., Quate C. (2001). Ultralow biased field emitter using single-wall carbon nanotube directly grown onto silicon tip by thermal chemical vapor deposition. Appl. Phys. Lett..

[90] Han I.T., Kim H.J., Park Y.J., Lee N., Jang J.E., Kim J.W., Jung J.E., Kim J.M. (2002). Fabrication and characterization of gated field emitter arrays with self-aligned carbon nanotubes grown by chemical vapor deposition. Appl. Phys. Lett..

[91] Wadhawan A., Stallcup R.E., Stephens K.F., Perez J.M., Akwani I.A. (2001). Effects of O_2_, Ar, and H_2_ gases on the field-emission properties of single-walled and multiwalled carbon nanotubes. Appl. Phys. Lett..

[92] Guillorn M.A., Hale M.D., Merkulov V.I., Simpson M.L., Eres G.Y., Cui H., Puretzky A.A., Geohegan D.B. (2003). Integrally gated carbon nanotube field emission cathodes produced by standard microfabrication techniques. J. Vac. Sci. Technol. B.

[93] Yang Q., Xiao C., Chen W., Singh A.K., Asai T., Hirose A. (2003). Growth mechanism and orientation control of well-aligned carbon nanotubes. Diam. Relat. Mater..

[94] Bocharov G.S., Eletskii A.V., Sommerer T.J. (2011). Optimization of the parameters of a carbon nanotube-based field emission cathode. Tech. Phys..

